# Overview of the Antioxidant and Anti-Inflammatory Activities of Selected Plant Compounds and Their Metal Ions Complexes

**DOI:** 10.3390/molecules26164886

**Published:** 2021-08-12

**Authors:** Paulina Mucha, Anna Skoczyńska, Magdalena Małecka, Paweł Hikisz, Elzbieta Budzisz

**Affiliations:** 1 Department of the Chemistry of Cosmetic Raw Materials, Faculty of Pharmacy, Medical University of Łódź, Muszyńskiego 1, 90-151 Łódź, Poland; 2 Department of Pharmacology, School of Pharmacy with the Division of Laboratory Medicine in Sosnowiec, Medical University of Silesia, Poniatowskiego 15, 41-200 Sosnowiec, Poland; anna_sko@onet.pl; 3 Department of Physical Chemistry, Faculty of Chemistry, University of Lodz, Pomorska 163/165, 90-236 Łódź, Poland; magdalena.malecka@chemia.uni.lodz.pl; 4 Department of Molecular Biophysics, Faculty of Biology and Environmental Protection, University of Lodz, Pomorska 141/143, 90-236 Łódź, Poland; pawel.hikisz@biol.uni.lodz.pl

**Keywords:** flavonoids, crystal structure of metal ion complexes with flavonoids, antioxidant, anti-inflamatory activity

## Abstract

Numerous plant compounds and their metal-ion complexes exert antioxidative, anti-inflammatory, anticancer, and other beneficial effects. This review highlights the different bioactivities of flavonoids, chromones, and coumarins and their metal-ions complexes due to different structural characteristics. In addition to insight into the most studied antioxidative properties of these compounds, the first part of the review provides a comprehensive overview of exogenous and endogenous sources of reactive oxygen and nitrogen species, oxidative stress-mediated damages of lipids and proteins, and on protective roles of antioxidant defense systems, including plant-derived antioxidants. Additionally, the review covers the anti-inflammatory and antimicrobial activities of flavonoids, chromones, coumarins and their metal-ion complexes which support its application in medicine, pharmacy, and cosmetology.

## 1. Introduction

Our health depends on well-functioning metabolic processes, homeostasis and optimally functioning repair mechanisms in our body. Free oxygen radicals play a major role in the proper functioning of our body [1,2,3], which in some cases can lead to oxidative stress, which is the cause of a number of dysfunctions in the human body. It is well-known that oxygen is essential for the respiratory process and the lack of this element is synonymous with death. Proper oxygen metabolism determines all our life processes [4]. However, oxygen also has a second face—it is a source of reactive oxygen/nitrogen species (ROS/RNS), which in excess can lead to serious health disorders [5,6].

Chemically, reactive oxygen/nitrogen species are molecules that have at least one unpaired electron [7]. In practise, this means that free radicals are extremely reactive molecules with short lifetimes that react chemically with cellular structures rapidly. Free radicals are constantly produced by all cells under physiological conditions and affect our bodies every day [8]. In homeostasis, ROS plays an important role in physiological processes while maintaining an appropriate concentration. These molecules are involved in bactericidal and bacteriostatic processes and act as messengers in cell signaling. They regulate the expression of certain genes, influence calcium metabolism and protein phosphorylation processes. They are also involved in muscle contractions, hormone production, and regulation of vascular tone.

Flavonoids, chromones and coumarins are groups of natural compounds found in fruits (citrus, blueberries, northern raspberries), vegetables (tomatoes, broccoli, peppers, lettuce, chicory, legumes, celery) as well as in numerous medicinal plants such as cinnamon, peppermint, green tea [9], higher plants and fungi from the families: Rutaceae, Apiaceae, Fabaceae, Rubiaceae, Hippocastanaceae, Solanaceae, Asteraceae, Poaceae, Aspergillus, Ceratocystis, Fusarium, Penicillinium, Streptomyces, Aloe, Aquilaria, Berchemia [10], Hypericum [11], Cassia, Artemisia, Dysoxylum, Ferula, Seseli, Pancratium, Peperomia, Ostericum, Nicotiana. All of them, as well as many of their metal ion complexes, are reported to possess antioxidant activity due to their chemical structure and specific substitution patterns, which include both inactivation of ROS and prevention of their formation.

The aim of this review is to show the diversity of biological properties of this type of plant compounds and to mention their metal ion complexes. In particular, their wide range of antioxidant, antimicrobial and anti-inflammatory effects is described, which allow them to prevent and alleviate the course of many diseases, often caused by the influence of reactive oxygen species on the human body.

## 2. Mechanism of Free Radicals

Research shows that the stationary concentration of reactive oxygen species and the reaction rate at which these molecules damage cell components depend strictly on the equilibrium level between the dynamics of ROS production in the body, and the concentration of low molecular weight antioxidants and the activity of protective enzymes. Under normal conditions, there is a balance between the formation of reactive oxygen species and their inactivation. The result of the imbalance between the amount of antioxidants and free radicals in the body is a pathological condition called oxidative stress. The increase in the rate of production of ROS/RNS in the body and the shift of the balance between pro-oxidant and antioxidant factors towards the “oxidation” reaction and, on the other hand, the lack or malfunction of factors that protect the body from ROS/RNS lead to an increase in the rate of free radical reactions. The result of oxidative stress is severe cellular damage and metabolic dysfunction, often leading to cell death and, in extreme cases, the development of cancer. The molecular mechanism of oxidative stress includes DNA damage and the formation of genetic mutations, oxidation of proteins and lipids of cell membranes, changes in the functioning of proteins and the induction of apoptosis. It has also been proven that oxidative stress accelerates the aging process of the body. It should be emphasized that low concentrations of reactive oxygen species in our body are not harmful. Too high a concentration can lead to many diseases, inflammation, and disruption of homeostasis [1,2,8].

The biological paradox of the function of ROS/RNS in cells and its role in cellular metabolism is a kind of double-edged sword. On the one hand, ROS/RNS play an important role as regulatory/protective molecules; on the other hand, they are highly toxic molecules whose activity underlies many serious diseases, mutations and neoplasms. As it turns out, this dualism of molecular activities is explained by the concentration of ROS/RNS in cells, which determines their mode of action. It is believed that the source of oxidative stress in humans can be many factors, primarily related to an unhealthy lifestyle. Usually, this condition results from excessive exposure of cells, tissues or organisms to additional sources of ROS/RNS or acceleration of the production rate of the endogenous pool of ROS/RNS. The main sources of free radicals and reactive oxygen species are undoubtedly the physiological metabolic processes required for the proper functioning of our body and the maintenance of homeostasis. However, a significant increase in ROS production in cells can occur as a result of excessive exposure to harmful external chemical or physical factors [3,4,9].

An important group of compounds with unpaired electrons characterized by high chemical activity are the reactive forms of nitrogen, which include nitric oxide (II) (NO) and the compounds formed from it as a result of metabolic changes: Nitrosonium cation (NO^+^), Ni-troxylanion (NO^−^) and peroxynitrite (ONOO^−^). Under physiological conditions, these compounds play a protective role for the body against microbes. When there are disturbances in the production of RNS, there is an excessive increase in their levels, leading to the occurrence of nitrosation stress, a phenomenon similar to oxidative stress. The consequences of nitrosation stress are the nitrosylation reaction of proteins, which alters their structure and inhibits their catalytic activity. Proteins containing transition metals in their structure are particularly sensitive to the action of RNS. As a result of the interaction of RNS with such polypeptides, hemoglobin, myoglobin, aconitase, and cytochrome c are altered. In addition, RNS lead to pathological changes in the organization of the cytoskeleton and cell signaling, and through interaction with lipids they lead to their Pe oxidation and changes in the fluidity of cell membranes [5,12].

### 2.1. Exogenous ROS Production

Exposure to environmental factors such as smoking, UV, heavy metals, ozone, allergens, drugs and toxins, environmental pollutants can lead to an increase in ROS in cells. As a result of ionizing radiation, water molecules are broken down, which is part of the organic structures of cells. As a result of the radiolysis process, the excited water molecules break down into hydrogen atoms and hydroxyl radicals. Then, as a result of the interaction between the radiolysis products, particles of hydrogen peroxide can be formed. Research indicates that exposure of fibroblast cells to ionizing radiation led to an increase in reactive oxygen species. It is worth noting that radiotherapy strategies are currently being used for novel and improved cancer treatments, including the use of radiation-dependent generation of reactive oxygen species in cancer cells.

Ultraviolet radiation also contributes to the generation of ROS by, inter alia, stimulating the activity of NADPH oxidase. The absorption of UV radiation leads to the ionization and disintegration of the molecules with the final formation of free radicals. The effects of UV radiation include the formation of ozone, which can affect lung function, causing inflammation in the epithelium of the respiratory [6,13,14].

### 2.2. Endogenous ROS/RNS Production

Mitochondria are the major site for the generation of the internal physiological pool of reactive oxygen species. As a result of oxygen metabolism, the transport of electrons in the mitochondrial chain (oxidative phosphorylation and ATP production) produces the superoxide radical. Normally, electrons for oxygen reduction are transferred through the mitochondria (ETC), but it is estimated that about 1–3% of electrons escape from this system and lead to the formation of superoxide through the process of one-electron reduction in oxygen [7]. So far, 11 sites in mitochondria have been identified where the production of superoxide radical anion takes place. The major elements responsible for electron loss and generation of ROS (superoxide radical) are NADH-CoQ oxidoreductase (EC 1.6.5.3) and ubiquinone. In complex I of the respiratory chain, ROS can be formed both during proper functioning of the respiratory chain and during reverse electron transport (against the redox potential of the electron transporters). The chain complex II can also generate a superoxide radical under hypoxia. In this situation, succinate dehydrogenase (EC 1.3.5.1) acts as a reducing agent, and the fumarate becomes an electron acceptor of ubiquinone. At a later stage, the superoxide anion can be converted to hydrogen peroxide in the mitochondrial matrix (MM) or in the intermembrane mitochondrial space (IMS) with the participation of enzymes-manganese superoxide dismutase (Mn-SOD) and Cu- or Zn-SOD. Moreover, the H2O2 in the IMS can be converted into a hydroxyl radical by a Fenton reaction [15,16,17,18].

As shown by numerous studies, mitochondrial dysfunction and increased formation of ROS in these organelles is characteristic of the etiology of many diseases, such as inflammation, neurodegenerative diseases, diabetes, and even cancer. The increased level of ROS results in damage to the nuclear structures that are central to the proper functioning of the cell, such as lipids, proteins, sugars, and especially DNA. The deleterious effect of ROS is also attributed to the activation of oncogenes with simultaneous inhibition of suppressor genes, leading to uncontrolled cell proliferation and promotion of tumour development [19,20,21,22].

It should be emphasized that mitochondria are also an important source of reactive forms of nitrogen and nitrogen radicals. The synthesis of nitric oxide is mainly mediated by inducible nitric oxide synthase type 2 (NOS2) in macrophages. Nitric oxide is a highly toxic, non-specific compound. In the cell, this compound has an affinity for metal proteins and can affect the process of gene transcription in the nucleus by activating certain transcription factors. Nitric oxide can be reduced to the nitroxide anion (NO^−^) or oxidized to the nitrosonium cation (NO^+^) in the presence of a strong oxidizing agent such as ^•^OH. NO^+^ can nitrosylate proteins and DNA and also reacts with the superoxide radical anion (O_2_^−^) to form peroxynitrite (ONOO^−^). Peroxynitrite is a highly reactive form of nitrogen that readily oxidizes glutathione, methionine, ascorbate, purines, and pyrimidines. It is capable of initiating the process of lipid peroxidation and inhibits the activity of I, II and IV of the respiratory chain complex. In addition, OONO^−^ reacts rapidly with CO_2_, leading to the formation of CO_3_^−^ and NO_2_^−^ radicals, which oxidize and nitrate proteins [23,24].

Another important source of the pool of ROS in cells is the process of phagocyte respiratory burst. It plays an important role in the body’s fight against microbes, viral infections and parasites. The key enzyme for the implementation of the respiratory burst is the enzyme NADPH oxidase, which is located on the inside of the plasma membrane of phagocytes. The action of NADPH oxidase is to transfer electrons from NADPH to molecular oxygen, resulting in the formation of a superoxide radical, a precursor to the subsequent forms of reactive oxygen species used in direct control of microbes and parasites. The superoxide anion radical is converted in phagocytes to hydrogen peroxide (with the participation of the enzyme superoxide dismutase), which in turn is converted to compounds with high bactericidal activity—e.g., hypochlorous acid, hydroxyl radical, chloramine-with the participation of myeloperoxidase. Of course, the activity of NADPH oxidase is subject to constant molecular control, which regulates and prevents excessive ROS production leading to oxidative stress [25].

Other organelles that can endogenously produce reactive oxygen species, particularly hydrogen peroxide, are peroxisomes. Biologically, peroxisomes are involved in many metabolic processes, such as activation and β-oxidation of fatty acids, participation in detoxification of the body (protection from, e.g., ethanol), hydrolysis and conversion of Ac-etylconezyme A, catabolism of amino acids, purines and polyamines. Remarkably, these organelles can both produce and degrade hydrogen peroxide because of the diversity of their enzymes: There is at least one flavin oxidase, the byproduct of the catalyzed reaction being H_2_O_2_, and catalase, which determines the process of decomposition of hydrogen peroxide into water and oxygen. Peroxisomes are also a source of superoxide anions, the production of which is caused by xanthine oxidase and the electron transport chain present in organelle membranes, which includes the flavoprotein NADH reductase and cytochrome b5. One of the electron acceptors is then the oxygen molecule [26,27,28].

The endoplasmic reticulum is also involved in the formation of the endogenous pool of ROS in cells. These organelles perform a variety of functions, including control of detoxification, protein folding, lipid metabolism, and calcium storage [29]. Molecular defects in the protein folding pathway lead to the accumulation of disordered proteins in the ER, which in turn disrupts homeostasis and triggers the formation of ROS in response to unfolded proteins. In newly synthesized proteins, oxidation of thiol groups and formation of disulfide bonds occur. The structural changes are catalyzed by enzymes present in the ER: Endoplazmatic Reticulum Oxidoreductin (ERO1 oxidoreductase) and protein disulfide isomerase (PDI isomerase), whose activity involves the transfer of electrons to molecular oxygen and the formation of H_2_O_2_. Studies show that overexpression of ERO1 and PDI is observed in various cancers and contributes to increased progression and metastasis, which is associated with shorter overall survival. Another site where ROS is generated on ER is the microsomal electron transport chain. Among the best known effects of this multicomponent enzyme system are cytochrome P450 and cytochrome b5. Among other things, these enzymes are involved in the detoxification of the body and catalyze the oxidation reactions of xenobiotics, as well as endogenous substrates (e.g., fatty acids). The result of the activity of these enzymes is the formation of a superoxide radical and H_2_O_2_ [30,31,32].

Generation of reactive oxygen species also occurs in the catabolic processes of thymidine [33]/polyamines [34] and in the autoxidation of reduced forms of low molecular weight cellular components-catecholamines, flavin nucleotides, thiol compounds, reducing sugars. In most cases, the by-product of metabolism is the formation of the radical anion Su-peroxide. There is evidence that the autoxidative processes of dopamine can lead to the death of neurons in the aging process of the body and in Parkinson’s disease. The superoxide anion radical is also produced in the one-electron oxidation reaction of hemoproteins-respiratory proteins involved in oxygen transport in the body. Autoxidation of hemoglobin leads to the formation of methemoglobin, which, in conjunction with the release of heme from MetHb, contributes to the development of inflammation. However, erythrocytes have a defense mechanism—the enzyme methemoglobin reductase, which maintains the appropriate redox state of these cells by reducing methemoglobin (Fe(III)) to hemoglobin (Fe(II)) [35].

Some enzymatic reactions may also be the source of the endogenous pool of reactive oxygen species. Endothelial Nox oxidases, despite many structural similarities with the oxidase found in phagocytes, are characterized by a completely different function. Because of their enzymatic activity, the nonphagocytic NADPH oxidases of the NOX family reduce molecular oxygen to the superoxide radical anion. However, the generated ROS are thought to be used in cells as signaling molecules in extracellular and intracellular signaling pathways [36].

### 2.3. Biological Outcomes of Oxidation by ROS

It is estimated that each cell is exposed to about 1.5 × 10^5^ oxidative influences per day due to the activity of ROS. Under conditions of homeostasis, the pool of exogenous or endogenous free radicals is inactivated by the action of antioxidants. As long as these are in balance, the body functions well. However, if for some reason there is an increase in free radical production or a decrease in antioxidant activity, a condition known as oxidative stress develops. Such a pathological condition can lead to the development of many diseases. Too many free radicals can damage virtually all cells and tissues. Unfortunately, free radicals attack molecules that are particularly important for the proper functioning of our cells, such as lipids, proteins and carbohydrates. Moreover, the destructive action of ROS leads to DNA mutations and genetic instability [4,8,37].

#### 2.3.1. ROS and Lipids

The most common free radical reaction disturbing the proper functioning of cells in the chain lipid peroxidation consisting of the oxidation of polyunsaturated fatty acid residues that are part of the membrane phospholipids and lipoproteins. Non-enzymatic peroxidation of lipids is a three-step process, and the main radical damaging the lipids of cell membranes is the hydroxyl radical. In the first stage of initiation, the hydrogen atom is detached from the unsaturated fatty acid molecule of phospholipids and alkyl radicals are formed (L^•^). The reaction results in the rearrangement of double bonds and the formation of conjugated bonds. In the next stage, propagation-free alkyl radicals may react with oxygen or fatty acids, causing the formation of further alkyl radicals or lipid peroxyl radicals (LOO^•^). It is a chain reaction that can lead to the autoxidation of several hundred molecules of polyunsaturated fatty acids. The last step is the termination of peroxidation, which can occur in a disproportionation reaction of two lipid alkyl/peroxyl radicals or two different radicals [38,39].

The products of the lipid peroxidation reaction are fatty acid dimers and oxo/hydroxy fatty acids with a modified and damaged structure. Further transformations of these peroxidation products lead to the formation of final, highly reactive aldehydes and hydroxyaldehydes, including malondialdehyde (MDA) and 4-hydroxy-2-nonenal (4-HNE). These compounds are treated as secondary lipid peroxidation transmitters that react with amino acid residues of proteins, disrupting their structure and function. It is particularly important for signaling pathways, where the conformational change in proteins dependent on MDA or 4-HNE often causes inhibition or induction of the activity of many key enzymes in the pathway. Moreover, MDA and 4-HNE interact with the nitrogenous bases of DNA causing chain damage which results in inhibition of DNA replication and [40,41,42].

These highly reactive lipid oxidation products have been shown to reduce antioxidant capacity and inhibit the activity of antioxidant enzymes. Moreover, α and β unsaturated aldehydes, by inhibiting or inducing the activity of many enzymes, are also involved in many pathways of signaling between cells or regulation of metabolic processes, contributing to their disturbances. Mitochondria are particularly vulnerable to lipid peroxidation. Oxidative changes in mitochondrial membrane phospholipids disrupt the electron transport chain and lead to increased production of ROS [43,44].

#### 2.3.2. ROS and Proteins

Apart from lipids membranes, structural and enzymatic proteins are exposed to the harmful effects of reactive oxygen species. The main cause of protein oxidation is the most reactive hydroxyl radical, although some modifications of proteins, such as the oxidation of thiol groups, may take place with the participation of superoxide anion and hydrogen peroxide. The process of protein oxidation with the participation of ROS concerns the polypeptide chain and amino acid residues and resembles lipid peroxidation; however, usually it is not a chain reaction. The oxidative activity of free radicals towards proteins includes many unfavorable changes in their structure: hydroxylation of aromatic and aliphatic amino acid residues, formation of protein hydroperoxides, oxidation of thiol groups and methionine residues, conversion of some amino acid residues into carbonyl derivatives, fragmentation of the polypeptide chain or creation of cross-links within the same or more polypeptide chains. The initiation of the oxidation process of the polypeptide chain takes place with the participation of the hydroxyl radical. It causes the detachment of the hydrogen atom on the α carbon of the amino acid. The resulting alkyl radical reacts violently with oxygen to form an alkyl hydroperoxide. This product can then be transformed into an alkoxy radical, which is directly responsible for the fragmentation of the polypeptide chain. The alkyl, alkyl peroxide, and alkoxy radicals may react with other amino acid residues of the same or a different polypeptide chain of the protein, allowing formation of further radicals. Presently, it is well-known that chemical modifications of proteins due to oxidation by reactive oxygen species can occur at almost any amino acid residue. The most susceptible to the harmful effects of free radicals, however, are cysteine and methionine containing thiol groups, and the aromatic amino acids tyrosine and tryptophan. Protein modification resulting from the action of reactive oxygen species has been shown to take place in the pathogenesis of many diseases and during the aging process. The strategic amino acids of enzymatic and regulatory proteins are often damaged. The most common consequence of structural changes is the loss of the biological functions of a protein, which may lead to inhibition of the activity of key enzymes or disorders of proteins with regulatory function during gene expression [45,46,47,48]. It has been shown that the increase in ROS-dependent protein peroxidation is correlated with the age of the organism. It is worth emphasizing, however, that increasing the level of oxidatively damaged proteins is also characteristic of many age-related diseases. Modified proteins are much less prone to proteolytic degradation, and as a result they accumulate in cells, often leading to necrosis [49,50]. Numerous studies also indicate that oxidative damage to proteins and the accumulation of their oxidized products play an extremely important role in the pathogenesis of cardiovascular diseases [51], especially in atherosclerosis and diabetes, as well as neurodegenerative diseases-Alzheimer, Parkinson. In diabetics with atherosclerotic complications, in the intravascular deposits, apart from oxidized lipoprotein LDL fractions, oxidatively modified proteins were also detected. Free radicals play a particularly large role in the etiology of neurodegenerative diseases. Neurons are particularly sensitive to oxidative disorders due to increased oxygen metabolism and high content of unsaturated fatty acids. The combination of unusually high metabolic and aerobic activity in brain cells is dangerous for proteins. It has been shown that many factors are involved in the pathogenesis of neurodegeneration, such as misfolded proteins and their aggregation, disturbances in signaling pathways caused by the action of reactive oxygen species [52,53,54].

#### 2.3.3. ROS and DNA

Nucleic acids are characterized by greater stability and resistance to free radicals. Oxidative damage to these cellular structures is repaired relatively quickly, and damaged nucleobases are excised from the DNA strand and degraded. The greatest threat to our DNA is the hydroxyl radical, which can cause free radical damage within virtually every nucleotide fragment. It is possible to oxidize nitrogen bases, sugar residues and phosphodiester bonding, leading to nucleotide modification, DNA damage and even strand breakage. Thymidine is particularly susceptible to the action of the hydroxyl radical, as it is damaged and transforms into thymidine dimers and peroxides. The reaction of guanine with the hydroxyl radical leading to the formation of 8-hydroxyguanine is also very harmful. Such a modification of the nitrogen base leads to the G-C- > T-A transversion, directly translating into errors in gene expression. It should be noted that the effect of reactive oxygen species on DNA, in addition to the modification of nitrogen bases, deletions or mutations, are also very often disorders of the association of transcription factors, increased expression of proto-oncogenes, chromosome breaks and a number of other anomalies that are lethal to cells [55,56,57]. Mitochondrial DNA, which does not contain intron sequences and is not protected by histones, is particularly vulnerable to RFT. Mitochondrial DNA has been shown to be much more susceptible to free radical damage than nuclear DNA, even during endogenous ROS generation. This fact is of particular importance for the etiology of degenerative diseases, where, as research shows, mitochondrial dysfunction caused by oxidative stress is found in most cases [58,59].

## 3. Antioxidant Defense System

The evolution of living organisms on Earth is inherently related to the environment rich in oxygen, and therefore also the permanent exposure of living organisms to the action of reactive oxygen species as by-products of oxygen metabolism. A necessary condition for the proper development and maintenance of homeostasis was the creation of efficient and effective mechanisms that would reduce the level of reactive oxygen species generated, ensuring their appropriately low level [60,61,62]. This system protects cells against ROS, neutralizing their pro-oxidative activity and at the same time ensures their appropriately low, physiological level. Antioxidant protection system works generally on three levels: (i) preventing the formation of free radicals (e.g., antioxidant enzymes and transition group metal ion-binding proteins) (ii) capturing and neutralizing (scavenging) ROS by antioxidants and (iii) repairing molecules damaged by ROS by repair enzymes, e.g., superoxide dismutase. Due to the complexity of the reactions and the environment of activity, antioxidants constitute a diverse group of compounds with a different chemical structure. Both hydrophilic and lipophilic compounds are among the antioxidants. Vege-tables and fruits are good sources of hydrophilic antioxidants. Vitamins belonging to this group of compounds, due to the water environment, are relatively easily absorbed by our body. On the other hand, readers should keep in mind that they are quickly cleared from the body in the urine. However, it should be emphasized that some polyphenols, especially flavonoids, are characterized by poor bioavailability. Their absorption in the intestine is low. Lipophilic antioxidants are absorbed in the presence of fats, so the body can use them only in their presence. The undoubted advantage of this group of antioxidants is the fact that they are more difficult to remove from the body, so they perform their antioxidant functions for longer. From the point of view of the source of antioxidants in the body, we can divide them into exogenous antioxidants—they are supplied only with food, and endogenous ones, which our body is able to synthesize itself. The second type of division of free radical scavengers is the way they perform their biological functions-there are enzymatic and non-enzymatic antioxidants. The most commonly used and the most typical, however, is the division of antioxidants into two groups. The first is the so-called first line of defense against ROS, which includes primary and secondary enzymes. The secondary enzymes play an indirect role in the removal of ROS by supporting endogenous antioxidants. The second group of antioxidants are non-enzymatic, low-molecular compounds that protect against the formation of ROS by direct reaction with them or with indirect metabolites of the redox reaction. Antioxidants from this group are active in the hydrophilic (ascorbic acid) as well as the hydrophobic (tocopherols, carotenoids) phase. The vast majority of non-enzymatic antioxidants, such as vitamins, bioflavonoids or carotenoids, unfortunately, are not synthesized by our body, which is why their proper supplementation with an appropriate diet is so important [4,63,64].

Primary enzymes, as donors of free hydrogen atoms, react directly with free radicals, interrupting free radical chain reactions. The main enzymes that remove ROS are superoxide dismutase, catalase and glutathione peroxidase.

Superoxide dismutase (SOD E.C. 1.15.1.1) is a metalloenzyme that catalyzes the dissolution of a superoxide radical to hydrogen peroxide and molecular oxygen. SOD superoxide dismutase is an extremely important enzyme in the protection of mitochondria and the fight against oxidative stress. There are three isoforms of superoxide dismutases in human cells: SOD1 copper-zinc, found mainly in the cytoplasm, SOD2 manganese, which is located in the mitochondria, and extracellular superoxide dismutase SOD3, which also contains copper and zinc, but is located within cell membranes and the intercellular matrix. SOD2 is often considered the most important form of SOD in humans, especially in the brain. Mutations in the SOD2 gene have been shown to be associated with diseases such as cardiomyopathy and motor neuron disease. Low activity of this enzyme has been linked to stroke, Alzheimer’s and Parkinson’s disease [65,66,67].

The hydrogen peroxide generated by the action of SOD can then be broken down by another important antioxidant enzyme, catalase (CAT, EC 1.11.1.6). CAT is a protein enzyme, a type of hemoprotein that is localized in the mitochondria of cells as well as in the peroxisomes. It plays a significant role in protecting cells against ROS and has antioxidant properties. It is one of the most effective protective enzymes. One molecule of catalase can convert millions of molecules of hydrogen peroxide into water and oxygen every second. This enzyme has dual activity: catalase and peroxidase. The primary function of catalase is participation in the disproportionation of hydrogen peroxide to produce oxygen and water. Catalase exhibits peroxidase activity in relation to some chemical compounds. It catalyzes the oxidation reaction of ethanol, methanol, formate, nitrites, quinones and others. Catalase is therefore an enzyme that functions both in the catabolism of hydrogen peroxide and in the oxidation of exogenous substrates. It is believed to play a special role in inflammation, mutagenesis and carcinogenesis [65]. The reduction in catalase activity accompanies many diseases, incl. cancer, neurodegenerative diseases (Parkinson’s disease, Alzheimer’s disease), inflammatory bowel diseases [68].

Another enzyme that is part of the primary protective enzymes against ROS is glutathione peroxidase (GPX, EC 1.11.1.9), which reduces hydrogen peroxide to water and maintains the correct concentration of glutathione in cells thanks to the ability to convert oxidized glutathione (glutathione disulfide) into its reduced form GSH. The enzyme is found mainly in the cytosol and mitochondria, where it accounts for about 20% of total activity, and in the cell nucleus. It belongs to the selenoperoxidases containing selenium in the form of selenocysteine in the active center. GPX requires the presence of selenium and glutathione to be effective in antioxidant activity. Without them, this enzyme has reduced activity and cannot effectively protect us against free radicals. Low glutaionic peroxidase activity may thus be correlated with a deficiency of glutathione and/or selenium, possibly with mutations in the gene encoding this enzyme [69,70].

Enzymes belonging to the peroxiredoxin family (PRDX, E.C 1.11.1.1) are also important in scavenging free radicals. These enzymes are classified as peroxidases—oxidoreductases that catalyze the oxidation of hydrogen peroxide. So far, six different classes of PRDX have been identified, of which 1-Cys PRDX or 2-Cys PRDX shows redox-active cysteine residues. The catalytic enzymatic activity of PRDX is based on the decomposition of H_2_O_2_ and the subsequent regeneration of the enzyme by disulfide oxidoreductases, such as thioredoxin and glutathione [71].

The activity of all the above-mentioned enzymes is ensured, among others, due to the constant regeneration of the reduced form by reductants-mainly GSH and Trx. This is usually performed by some reductases, NADPH-dependent-E.C 1.8.1.7 glutathione reductase and E.C 1.8.1.9 thioredoxin reductase. It is worth noting that reduced NADPH is necessary for these reductases for their continued activity. Therefore, the enzymes whose biological activity is responsible for the continuous production of NADPH can be considered as secondary antioxidants, the misfunction of which can disrupt the entire ROS balance [72].

The second line of defense against antioxidants mainly consists of reduced thiols and low molecular weight antioxidants (LMW) soluble in both water and fats. The undoubted advantage of LMW is their ability to move within the system to places where reactive oxygen species appear and damage resulting from their activity. Endogenous LMW includes, inter alia, glutathione [35].

An important group of low molecular weight antioxidants are thiols, which can react with most physiological oxidants. Tiols maintain a homeostatic intracellular and tissue redox status. One of the most important low molecular weight thiol antioxidants is the glutathione tripeptide. It is found primarily in the cytosol, but also in the peroxisomes, mitochondria and the cell nucleus. The ability to perform antioxidant functions is due to the high concentration of glutathione in our cells, from 5 to 10 mM, but also the specific structure—the characteristic isopeptide bond and the presence of the SH group belonging to the cysteine residue. The thiol group is very easy to react with free radicals, as well as with free radicals of organic substances, contributing to their biological inactivation and repair, respectively [73,74].

High ability to chelate heavy metal ions is also demonstrated by metallothioneins (MT), low-molecular proteins containing numerous cysteine residues, involved in the detoxification of organisms from harmful metal ions and in the defense, reaction associated with oxidative stress. MT located in the membranes of Golgi apparatus exhibit strong antioxidant properties and protect cell structures against free radicals, especially reactive oxygen species. In humans, they play an important role in the metabolism of zinc. Due to this function, they are associated with aging, civilization diseases and cancer [75,76].

Coenzyme Q10 is another endogenous molecule with antioxidant functions. This isoprenoid free radical scavenger is lipid soluble found in cell membranes and its antioxidant activity is particularly important for the mitochondrial electron transport chain. Moreover, Q10 protects membrane lipids and lipoproteins from peroxidation and oxidative damage. In its active form (quinol), Q10 can also regenerate volatile antioxidants-including vitamins C and E [77,78].

In addition to endogenous, dietary antioxidants such as vitamins contribute to significant protection against reactive forms of oxygen-water-soluble vitamin C and fat-soluble vitamin E. Vitamin C occurs in two redox forms: reduced ascorbic acid (AA) and dehydroascorbic acid (DHA). DHA can be regenerated as a result of GHS or Trx related mechanisms. Ascorbic acid functions in our cells as an effective antioxidant. This function is carried out both directly and indirectly through the regeneration of oxidized vitamin E, GSH or carotenoids. Directly, ascorbic acid protects lipids against peroxidation, which is of particular importance for brain tissues. The radicals directly scavenged by AA include, among others, the hydroxyl radical, superoxide anion, hydrogen peroxide [79,80]. Vitamin C, as one of the strongest natural antioxidants, also performs its biological activity as a free radical scavenger in the skin. It prevents free radical reactions in the skin resulting from the action of UV radiation, contributing to the photoprotection of the skin, preventing its photoaging and the development of photocarcinogenesis. The anti-aging activity of vitamin C results, among others, from its participation in the synthesis of collagen, stabilization of collagen fibers and inhibition of their degradation. Vitamin C is a cofactor of key enzymes for the cross-linking and stabilization of collagen fibers-prolyl and lysyl hydroxylase. Furthermore, this antioxidant has been shown to regulate the biological activity of transcription factors involved in collagen synthesis and to stabilize procollagen mRNA that regulates type I and III collagen synthesis. Vitamin C-dependent inhibition of AP-1 is observed, which in turn leads to a reduction in the expression of collagen-degrading matrix metalloproteinases (MMPs), reduces collagen production and increases elastin accumulation. It is also extremely important that vitamin C increases the expression of the collagen gene [81].

In the case of natural vitamin E, the most biologically active form is α tocopherol. Vitamin E protects lipids against the damaging effects of free radicals and their peroxidation by interrupting the chain reactions accompanying this process. It provides a hydrogen atom to the lipid radicals, lipoxyl and peroxyl, with the formation of lipids, alcohols and hydroxy peroxides, respectively. Due to its lipophilic nature and place of action (cell membranes), vitamin E also contributes to the protection of low-density lipoprotein against free radical damage [82,83].

Another example of effective lipid-soluble antioxidants is the complex group of compounds known as carotenoids. One of the most common compounds of this group is the provitamin A precursor-β carotene. This compound, as a strong antioxidant, takes part in scavenging singlet oxygen. Its antioxidant activity is comparable to α tocopherol. The process of quenching singlet oxygen consists of absorbing energy by the structure of β carotene rich in electrons (presence of double bonds), which in the next stage is released in the form of heat. During this process, the structure of the antioxidant does not change [84,85].

When discussing exogenous antioxidants, it is absolutely necessary to mention an extremely complex group of compounds widely distributed in the plant world: polyphenols. They belong to the plant secondary metabolites with different biological properties. In terms of chemical structure, polyphenols are characterized by the presence of one or more aromatic rings in a molecule and a different number of hydroxyl groups, which determines their biological activity. Polyphenols have antibacterial and anti-inflammatory properties and are an important component of the diet. It has been suggested that they may reduce the risk of cardiovascular disease and cancer formation. It has been shown that polyphenols as a very diverse and complex group of compounds are characterized by multiple antioxidant activity. These compounds, as free radical scavengers, effectively inhibit the activity of superoxide anions, H_2_O_2_, lipid peroxides, and the hydroxyl radical. In addition, some flavonoids are able to chelate metal ions preventing the Fenton reaction and blocking free radical generation [1].

Of course, the above-mentioned antioxidants are only examples of the most active compounds and the nature of free radical scavengers. It should be mentioned the presence of other endogenous antioxidants in the human body. This group includes, among others, uric acid, bilirubin and ceruloplasmin. These compounds, acting in specific places in cells, scavenge free radicals, contributing, inter alia, to the inhibition of lipid peroxidation, protect against mitochondrial oxidative stress and ensure iron homeostasis [35].

## 4. Biological Activities of Plant Compounds

Antioxidants are a group of biologically active compounds whose primary function is to protect cells and tissues of a living organism against the harmful effects of free oxygen radicals. Antioxidants interact with free radicals and are responsible for non-specific reactions that ultimately inactivate the reactive oxygen species. In recent years, more and more has been said about the high benefits for health of including foods rich in antioxidants in one’s diet. Nutritionists and scientist encourage to enrich your daily diet with them, and cosmetologists recommend cosmetics rich in these compounds. Nowadays, due to the wide range of biological properties in the human body, antioxidants play an important role in the prevention of civilization diseases, treatment of cancer, and show microbiological properties [1,86]. Below, we present a summary of the most important applications of antioxidants in medicine as compounds with potential anticancer, antibacterial, anti-inflammatory and anti-diabetic properties.

### 4.1. Anticancer Activity

Cancer is currently the most common cause of death, apart from cardiovascular diseases. Despite the dynamically developing chemotherapy, the synthesis of newer and newer compounds with potential anticancer properties, the adequately high selectivity of their action remains a big problem. The available chemotherapeutic agents are often associated with high side effects and systemic toxicity. High hopes are connected with natural compounds that are intermediate metabolites of plants, such as polyphenols.

In a number of in vitro and in vivo studies, it has been shown that this complex group of compounds exhibits antitumor properties by participating in a number of cell signaling pathways, implementing an apoptotic program or autophagy. The anticancer potential of polyphenols may result from the extremely high chemical diversity. The polyphenol compounds used in the research include a large number of plant extracts rich in polyphenols and isolated pure compounds [87,88,89,90,91]. Such a variety of compounds and the diversion of their molecular activities mean that they can be used in the treatment of almost any type of cancer, including cases of multi-drug resistance [92]. In vitro and in vivo studies suggest that polyphenols from various food sources may play a key role in delaying the development and progression of cancer by reducing cell proliferation, inactivating carcinogens, inhibiting angiogenesis, inducing cell cycle inhibition and apoptosis, and modulating the immune response [93,94,95].

Undoubtedly, one of the mechanisms of polyphenols responsible for their anticancer properties is the control of ROS homeostasis. Polyphenols show a dual action in relation to ROS homeostasis. Under physiological conditions, they function as antioxidants, but in cancer cells they can indirectly suppress pro-oxidative enzymes and contribute to the generation of reactive oxygen species in cancer cells, leading to their apoptosis. Numerous studies indicate that just such a double action of polyphenols in the area of ROS level modulation is one of their anti-tumor, pro-apoptotic and anti-proliferative mechanisms of action [96,97]. The pro-oxidative properties of polyphenols are often associated with the induction of morphological changes in cancer cells characteristic of apoptosis and DNA damage. Jin et al. [98] showed that daidzein induces MCF-7 breast cancer cell apoptosis via the mitochondrial pathway due to the ROS generation. Similar results were also found in recent studies in the case of hesperidin and naringenin, which induced the programmed death pathway of cancer cells of various lines by increasing the production of ROS and activation of signaling pathway [99,100,101]. Another polyphenol, flavonol kaempferol inhibited the growth of bladder cancer cells by inducing apoptosis induced by ROS level modulation and S phase arrest [102]. Changes in ROS levels in neoplastic cells induced executive apoptosis caspases in HCT116, HCT15 and SW480 colon cancer lines [103]. In addition, kaempferol exerted a cytotoxic effect on rat hepatocellular carcinoma cells through ROS mediated mitochondrial targeting [104].

The antitumor abilities of antioxidants result primarily from the ability of these compounds to activate apoptotic pathways in neoplastic cells or autophagy. This skill is realized on many levels of the molecular machinery involved in this process. Research shows that polyphenols are capable of suppressing the proliferation of neoplastic cells by inhibiting a number of cell pathways that promote cell division. Recent studies indicate that flavonoids are capable of inducing cell cycle arrest in the G2/M phase of cancer cells by both suppressing signaling from the EGFR/Mitogen Activated Protein Kinase (MAPK) pathway, which was responsible for apoptosis and cell cycle arrest [105,106], as well as increasing the expression of genes encoding cell cycle regulatory proteins-cyclin-dependent kinase inhibitor-p21 [107]. The studies by Zhang et al. [108] provide very interesting results on the effect of flavonoids from *Tephroseris kirilowii* (Turcz.) Holub, which in breast cancer cells caused downregulation of PI3Kγ-p110 and consequent interruption of PI3K/AKT/mTOR/p70S6K signaling pathway. The effect of flavonoids was the inhibition of the cell cycle and the activation of both apoptosis and autophagy in cancer cells. Modulating the expression of genes involved in cell proliferation with the participation of antioxidants may also proceed through the influence of these compounds on the activity of the NF-κB transcription factor. Anthocyanins/anthocyanidins inhibit the pro-inflammatory NF-κB pathway, attenuate Wnt signaling and suppress abnormal colorectal cancer cell proliferation have been shown [109]. The pro-apoptotic activity of antioxidants is obviously related to their ability to influence the expression of genes both coding for proteins that are crucial for the process of programmed death, and of an anti-apoptotic nature (e.g., surviving). A number of studies indicate that this complex group of compounds in cancer cells induces the expression of genes whose protein products are directly involved in apoptotic pathways with simultaneous inhibiting anti-apoptotic genes. Antioxidants are able to induce an increase in the expression of pro-apoptotic proteins of the Bcl-2 family: Bax, Bak and Bid, while decreasing the activity and expression of their anti-apoptotic partner-Bcl2, Bcl-xL. In addition, antioxidants increase the activity of the key initiator caspases 8 and 9 of the external and internal pathways in tumor cells, and the effector caspase 3 of programmed cell death [110,111,112,113].

It is also extremely important that, as reported by Khan et al. the anticancer effects of antioxidants may also result from their impact on one of the key signaling pathways involved in tumor suppression, involving the tumor suppression of the p53 protein. A number of polyphenols, including curcumin, resveratrol, genisteine, quercetin, wogonin and epigallocatechin, increase p53 expression in neoplastic cells. Moreover, it is suggested that polyphenols can stabilize p53 protein by controlling its phosphorylation/acetylation processes and reduction in oxidative stress [114]. Recent research indicates an innovative approach to the use of polyphenols in anticancer therapy. It tries to use nanotechnologies to improve the solubility and bioavailability of polyphenols [115]. Devi et al. point to the use of polyphenols in cancer treatment through miRNA regulation [116].

### 4.2. Anti-Inflammatory Activity

The immune system is a very important part of human body because it allows us to function in the surrounding environment. It is the guardian of immunity and prevents the development of various types of infections, detects and fights pathogens attacking the body, such as bacteria, viruses, parasites, fungi and toxins, and also takes part in fighting cancer, which is why its proper functioning is so important for health [117]. Chronic inflammation can cause many diseases, including atherosclerosis, irritable bowel syndrome, arthritis, cancer, and even depression. It is usually caused by an abnormal reaction of the immune system. Numerous studies show that polyphenols modulate the immune response of our body. The ability to influence the immune system is realized on several levels of molecular activity. Antioxidants can affect the expression of transcription factors involved in the immune response, induce the suppression of pro-inflammatory cytokines, inhibit key signaling pathways and enzymes involved in immune processes [5,118].

In the inflammatory process, cytokines play a key role as mediators in the body’s immune response. It is believed that the relationship between pro-inflammatory and anti-inflammatory cytokinins is an extremely important parameter determining the correct immune response during homeostasis and inflammation associated with diseases [119]. A number of studies indicate that the immunomodulatory effect of polyphenols is based on antioxidant control of the balance between pro-and anti-inflammatory cytokines. Quercetin and catechins increase the release of anti-inflammatory IL-10 with simultaneous inhibition of the pro-inflammatory cytokines TNF-α and IL-1Β [120]. Similar effects are shown by polyphenolic extracts from *Cydonia oblonga* [121]. Studies with the use of extra virgin olive oil or blueberry extracts showed that the polyphenols contained in these substances effectively counteracted the induction of inflammation in the subjects by inhibiting the secretion of pro-inflammatory cytokines IL-6, IL-12 [122,123]. Several studies using various types of cells, including murine models of LPS-induced inflammation, as well as human mast line cells, peripheral blood mononuclear cells, and astrocytes, have shown that one of the most common mechanisms of polyphenols used by which they exert their immunomodulatory effects is the inhibition of the expression of a number of cytokines pro-inflammatory: TNF-α, IL-1B, IL-6, IL-8 [124,125,126,127].

The immunomodulatory capacity of antioxidants is also manifested in the ability of these molecules to influence key signaling pathways responsible for inflammation. The expression of a significant number of genes directly involved in inflammation is controlled by NF-kB. Research shows that some of the polyphenols show the ability to modulate the activity of NF-kB and reduce inflammation in cells. The result of antioxidant activity is the inhibition of the NF-kB transcription factor, which in turn leads to the suppression of the expression of genes whose protein products act proinflammation [128,129]. Suppression of NF-kB dependent on the action of polyphenolic compounds and further inhibition of chronic inflammation in neoplastic cells turns out to be of key importance for tumor development and proliferation [130,131].

Another important pathway involved in a number of fundamental cellular processes such as proliferation, differentiation, cell death, and regulation of the expression of proteins involved in inflammation is the mitogen-activated MAPK kinase pathway. It is indicated that due to their chemical complexity, antioxidants may influence the MAPK pathway and regulate its action at many stages of the signal transduction pathway. Luteoin is able to reduce TNF-α release and inhibit ERK, JNK and P38 kinases in both LPS-induced mouse cells and human macrophages [132,133]. Quercentin also shows a similar biological effect, inhibiting the phosphorylation and activation of the above-mentioned kinases, leading to a reduction in TNF-α transcription and expression [134]. Several studies show that such an inhibitory effect of polyphenolic compounds on the MAPK kinase pathway in neoplasms contributes to the reduction in inflammation accompanying the proliferation of neoplastic cells, which in turn translates directly into the inhibition of the proliferation of neoplastic cells [135,136,137].

The ability of antioxidants to reduce the release of arachidonic acid, prostaglandins and leukotrienes is considered to be one of the most important functions in modulating the immune response and anti-inflammatory properties. The biological activity of polyphenols on the arachidonic acid signaling pathway and modulation of the body’s immune response is primarily achieved by the ability of antioxidants to inhibit key enzymes for this pathway: COX, LOX and PLA2. The activity of these enzymes is correlated with the metabolism of arachidonic acid and the production of inflammatory mediators-prostaglandins, thromboxane A2 and leukotrienes. Recent in vitro and in vivo studies indicate that certain polyphenols inhibit the expression of key enzymes of the arachidone pathway, which was directly related to the reduction in post-inflammatory cytokine secretion and transcription factors responsible for the activation of genes involved in a strong immune response. Moreover, it is worth emphasizing that such anti-inflammatory effects of flavonoids were an important factor in increasing the anti-tumor activity of cells of the immune system [138,139,140].

### 4.3. Antidiabetic Activity

Diabetes belongs to the group of so-called diseases of civilization that are currently an extremely serious global health problem. Once associated with being a disease of seniors, today it affects younger and younger people, even schoolchildren. Diabetes mellitus is a chronic disease caused by an impaired insulin secretion. Too little insulin in the body disrupts the use of glucose by the body’s cells, which causes the level of glucose in the blood to rise (hyperglycaemia). It is well-established that T2D results from insulin resistance in insulin sensitive tissues and subsequent pancreatic β-cell dysfunction. Currently, a growing number of studies indicate the effective use of polyphenol antioxidants in the treatment of type 2 diabetes. These compounds, due to their excellent properties of free radical scavengers, modulation of intracellular signaling pathways involved in glucose and insulin metabolism and anti-inflammatory properties, turn out to be an extremely attractive hope and future in the treatment of diabetes [141,142].

One of the serious factors disrupting the normal insulin secretion pathway and the loss of the function of the β cells of the langerhans islets is oxidative stress. However, research shows that various polyphenolic compounds neutralize oxidative stress and modulate the expression of relevant genes involved in both inflammatory and apoptotic processes, and insulin secretion. One of the strategies of antioxidant activity in the treatment of diabetes is the influence of these compounds on the modulation of the expression of genes crucial for the dysfunction/proper functioning of pancreatic β cells and free radical metabolism [141].

Numerous scientific studies indicate that polyphenols such as resveratrol [143,144], catechins [145], polyphenolic extracts of olive oil and brown rice [146], jojoba seed extracts [147] or epigallocatechin [148] contribute to the reduction in blood glucose, the protection of cells against oxidative stress and the increase in insulin activity and secretion by pancreatic β cells. These compounds, through their antioxidant activity, contribute to the reduction in the risk of coronary heart disease by preventing low-density lipoprotein oxidation, oxidative stress and cell damage. The strategy of protecting β cells is related to the ability of the above-mentioned compounds to modulate the expression of key genes for the proper functioning of β cells and insulin secretion such as Glut2, Glut4, mitochondrial transcription factor (Tfam), pancreatic and duodenal homebox 1 (Pdx1), glucokinase (GK) and insulin 1 (Ins1). In addition, antioxidants increase the expression of genes responsible for mitochondrial complex biogenesis such as nuclear factor erythroid 2-related factor (Nrf2) and Nrf1 or decrease in the expression of the p22phox gene activating NADPH oxidase, generating excessive ROS. Moreover, the diabetes treatment effect of these natural compounds is associated with the modulation of the expression of genes involved in stress and the protection of β cells from ER stress-induced apoptosis. It has been shown that these compounds can inhibit the phosphorylation of the c-Jun N kinase (JNK) pathway, affect the Forkhead box O1 (FOXO1) transcription factors or the kinase B (Akt) pathway, contributing to the extension of β-cell viability and insulin secretion.

In addition to modulating the expression of genes directly involved in the metabolism of free radicals and the protection of pancreatic β cells, antioxidants also improve insulin signaling pathways. In this case, the diabetic-healing effect of polyphenolic compounds is primarily to regulate genes involved in regulating mitochondrial oxidation of fatty acids, improve cell sensitivity to insulin, and increase metabolism of glucose uptake. Generally speaking, with reference to the available research, an extremely important function of polyphenols is to modulate the increase in the expression of the GLUT4 gene. It is the main insulin-dependent glucose transporter in both muscle and adipose tissues, and its deficiencies are associated with insulin resistance in cells [149]. Numerous studies show that polyphenols effectively increase the expression of the GLUT4 gene, causing protein translocation from intracellular stores to the plasma membrane, thus inducing an increase in cell sensitivity to insulin, mitochondrial biogenesis and glucose uptake. Of course, in addition to the increase in GLUT4 expression, after treating cells with antioxidants, there is also an increase in a number of other regulatory genes whose proteins are directly involved in insulin signaling pathways, such as Mef2a, Nrf1, PI3K or SIRT1 [148,150,151,152].

One of the negative consequences of type II diabetes is the increase in the activity of the enzymes glucose-6-phosphatase (G6Pase) and phosphoenolpurivate carboxykinase (PEPCK), which directly increases gluconeogenesis and hyperglycemia [153]. It turns out that these two enzymes can also target the biological antidiabetic activity of antioxidants. It has been shown that polyphenolic compounds such as epigallocatechin [154], cinnamon [155], black rice extracts [156], resveratrol [157] or hesperidin [158] inhibit in vivo and in vitro hepatic glucose production by downregulating the expression of PEPCK and G6pase, which simultaneously meant a decrease in blood glucose levels and an increase in cell sensitivity to insulin.

It turns out that in addition to the ability to scavenge free radicals, also the immunomodulatory properties of polyphenolic compounds and inhibition of inflammation allow these compounds to effectively influence the treatment of diabetes. During chronic glucolipotoxicity, excessive production of ROS leads to oxidative damage to proteins and lipids and the development of inflammation. Oxidative stress accompanying diabetes contributes to the loss of B cell function, worsening insulin resistance and the development of a number of vascular complications. Research shows that the use of polyphenolic compounds in the treatment of diabetes inhibits key inflammatory pathways involving NF-kB and TNF-α and suppresses the activity of pro-inflammatory markers such as COX-2 and IL1B [159,160].

### 4.4. Antibacterial Activity

One of the important functions attributed to polyphenolic antioxidants is their participation in defense reactions against pathogenic microorganisms, bacteria or fungi. Particularly great interest in the microbiological use of polyphenols concerns their use in the food industry as bactericides for food pathogens, but also as supporting agents for antibiotics available in medicine. Currently, a large part of the research is devoted to the microbiological properties of a number of different polyphenol extracts, both naturally occurring [161,162,163] and synthetic complexes [164,165]. The results show that polyphenol plant extracts often have attractive antibacterial properties against individual species of different bacterial strains such as streptococci, bacilli, staphylococci, and even much more [166,167,168].

It is worth emphasizing that the mechanisms of the antibacterial activity of polyphenolic antioxidants are still not fully explored and several possible pathways of molecular mechanisms are proposed. The extremely large chemical diversity of the tested antioxidants, as well as the studies conducted not on single, extracted substances, but on whole extracts, make it difficult to clearly identify the molecular mechanisms responsible for inhibiting the multiplication of microorganisms and their death. Recent studies indicate the relationship between the chemical structure and the antibacterial activity of antioxidants. It is believed that the key factor in modulating microbial properties is the number and position of hydroxyl and methoxyl groups in the antioxidant structure. A quantitative structure–activity relationship study showed that the microbial activity of polyphenols is related to the hydrophobic and amphiphilic character of the molecule and the OH group at position 3 of the C-ring [169,170]. Gram negative bacteria have been suggested to be generally more resistant to the effects of antioxidants. This may be due to the presence of a lipophilic outer membrane made of phospholipids in these bacteria, which makes their cell wall impermeable. Moreover, it is indicated that Gram-negative bacteria can chemically break down polyphenols by their enzymes [171].

One of the proposed mechanisms of antimicrobial action of antioxidants is the interaction with bacterial proteins present on their cell wall. The effect of the interaction is damage to bacterial membranes and their permeabilization, loss of chemiosmotic control, leakage of intracellular components, outflow of cytoplasmic components and eventual cell death [172,173]. Moreover, recent studies emphasize the inhibitory properties of polyphenols in relation to the production of biofilm by bacteria, which are achieved through suppression of quorum sensing and the movement of bacteria [174,175].

Interestingly, polyphenolic compounds commonly used as excellent antioxidants appear to induce endogenous oxidative stress in bacterial cells and lead to the formation of ROS. According to Xiong et. al, who tested the microbiological properties of epigallocatechin gallate against Escherichia coli, it was demonstrated that the ROS generated in bacterial cells are responsible for oxidative damage to fatty acids in bacterial membranes and ultimately lead to the death of pathogens [176]. Similar results confirming the pro-oxidative properties of polyphenols in relation to bacterial cells were obtained in other studies [177,178].

It turns out that the microbiological activity of polyphenols also results from the ability of these compounds to influence the biosynthesis of proteins that are key to metabolic processes and the proper functioning of bacteria. Changes in expression depending on the antioxidants of the respective bacterial proteins involved, for example, in energy metabolism and in the tricarboxylic acid cycle, DNA metabolism, and biosynthesis of fatty acids led to irreversible changes in their metabolism and final death [179,180]. Undoubtedly, an important property in the context of the antibacterial activity of polyphenolic compounds is their ability to inhibit the enzyme DNA gyrase, which in turn leads to inhibition of bacterial DNA synthesis [181]. Another enzyme crucial for the energy metabolism of bacteria is ATP synthase, which, as demonstrated by Chinnam et al. [182] and Dadi et al. [183], undergoes polyphenol-dependent inhibition resulting in the death of microorganisms.

## 5. Flavonoids, Coumarins and Chromones as Anti-Oxidative and Anti-Inflammatory Agents

### 5.1. Flavonoids as Antioxidants

The general structure of flavonoids consists of two benzene rings (A), (B) and a heterocyclic pyran ring (C), which is located next to ring (A). This class of compounds is divided into six subclasses, such as flavanols, flavonols, flavanones, flavones, isoflavones, and anthocyanidins [184].

Additionally, the presence of phenolic hydrogens enable them to act as molecules for hydrogen donating [185,186]. It is important for the regulation of inflammation, when phagocytes produce large amounts of oxygen and nitrogen derived radicals, which are released and have harmful effect on cell [187]. Flavonoids also prevent the formation of ROS, by chelating transition metal ions, mainly copper and iron, catalyzing ROS-forming reactions such as the hydroxyl radical [188,189]. Flavonoids possess the ability to inactivate oxygen radicals. The radicals that are most easily captured by flavonoids include superoxide anion radical, hydroxyl radical, singlet oxygen and lipid radicals [190]. Moreover, it was shown that flavonoids can reduce the activity of xanthine oxidase, which catalyzes the formation of superoxide radical anion [191]. It was found that 7-hydroxyflavones inhibit the activity of this enzyme competitively, but 3′,4′ or 3′,4′,5′-hydroxyflavones are non-competitive inhibitors [192]. Flavonoids also reduce the activity of the membrane oxidase NADPH, which is involved in the generation of superoxide anion [193]. Isoflavones, isoflavanones and isoflavates substituted with 6,7,4′-trihydroxy-and 6,7-dihydroxy groups show very high antioxidant activity, especially in the lipid phase. Flavanols and all catechins isolated from green tea, show the ability to capture hydrogen peroxide and superoxide radical anion generated in the xanthine-xanthine oxidase system [190].

**Figure 1:**
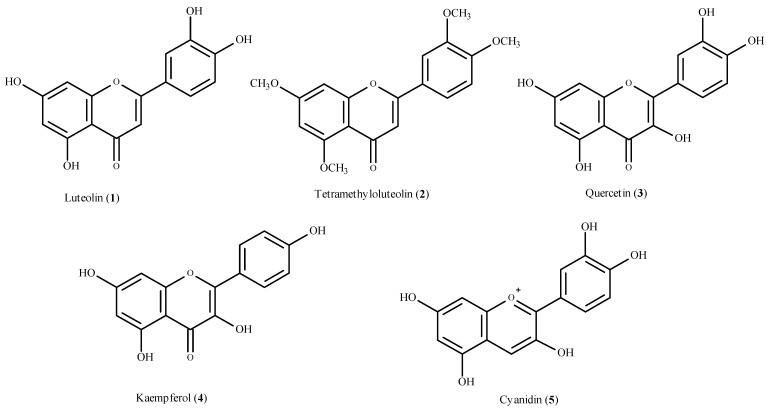
Structures of flavonoids mentioned in Section 5.1 with numbering.

By multiple mechanisms, flavonoids exert their anti-inflammatory properties. It is connected with suppression of CD80 and CD86, which are maturation markers and are important for CD4 + T cell activation, because of upregulation during maturation of dendritic cells (DCs). Moreover, flavonoids can modulate iron metabolism and that influences immune response of DCs [194]. Flavonoids can probably cause a decrease in release of histamine and prostaglandin from mast cells and suppress production of cytokines, chemokines in neutrophils [108,195,196,197,198]. Flavonoids can have an impact on cell signaling, because they can bind to the IL-17 receptor and attenuate its signaling. Oligomeric proanthocyanidins decrease expression of CD83 molecule and that inhibit maturation of pulmonary dendritic cells (DCs) [199]. Luteolin (**1**) (Figure 1) was found to decrease the release of histamine and prostaglandin D from mast cells [200]. Tetramethoxyluteolin (**2**) (Figure 1) showed stronger potential than luteolin (**1**). These two compounds suppressed the release of histamine, β-hexosaminidase and Tumor Necrosis Factor (TNF). Flavonols can act as anti-inflammatory agents, because they can inhibit cytokines release from mast cells and basophils [201]. It was revealed that quercetin (**3**) (Figure 1) reduced IL-4 and IL-5 cytokines and decreased accumulation of eosinophils in the bronchoalveolar fluid [202]. The reduction in allergic inflammation and decrease in cytokines involved NF-κB inhibition [202]. Kaempferol (**4**) (Figure 1) blocked degranulation of eosinophils in the lung tissue of murine model of allergic airway inflammation [201]. In the murine asthmatic model, genistein decreased inflammation of respiratory tract through suppression of transcription factors such as GATA-3 and STAT-6 [202]. Cyanidin (**5**) (Figure 1) binds to the interleukin 17 (IL-17) receptor and attenuates its signaling and it was observed in the mouse model of severe asthma, where cyanidin weakened inflammation induced by IL-17 [203].

### 5.2. Coumarins as Antioxidants

**Figure 2:**
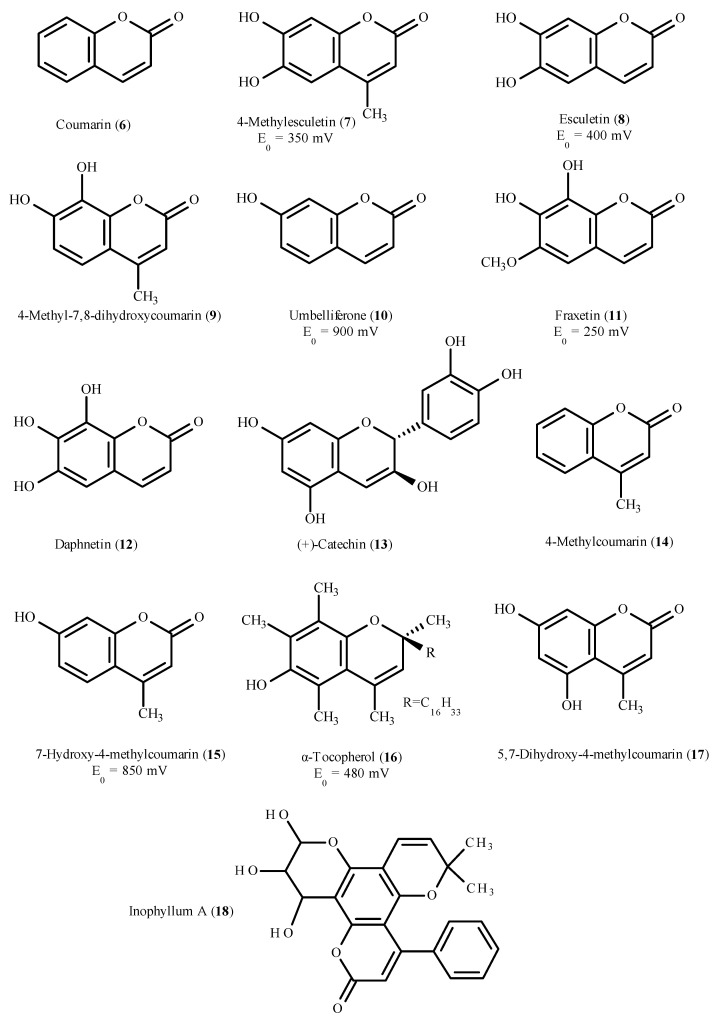
Structures of compounds mentioned in Section 5.2. and the oxidative potential (E_o_ [mV]) values of selected coumarins.

Coumarins are a diverse group of chemical compounds and are divided into four subtypes [204] simple coumarins, coumarins with a substituent on the pyrone ring, furanocoumarins, pyranocoumarins. Coumarins can exist as individual molecules or as aglycones in glycosides (β-*O*-glycosides) [205]. Coumarins with hydroxyl and methoxy substituents strongly absorb ultraviolet radiation and that allows them to be used as light protecting agents. In small doses, coumarins have relaxing effects, but in higher doses they exert a depressive effect on the central nervous system. Pyranocoumarins from *Ammi visnaga* (L.) exhibit antispasmolitic and vasodilating properties for blood vessels [205]. The main representant of this plant compounds is coumarin (**6**) (Figure 2), which has a sedative, anti-swelling, analgesic effect and spasmolytic properties in the digestive tract, coronary vessels and bronchi. It is used in the treatment and prevention of contractile states, spastic constipation, colic and neuroses [206]. For the first time, coumarin (**6**) was isolated from tonka bob (*Dipteryx odorata*) by Vogel in 1820 [207].

The in vitro and in vivo studies in rats indicated that the inhibition ability of coumarins is directly proportional to the number of hydroxyl groups in the molecule [208]. Antioxidant properties of coumarins manifest mainly in their ability to inhibit the production of reactive oxygen species (ROS). Coumarins affect xanthine oxidase, which is the enzyme involved in the generation of radical anion peroxides [209]. Coumarins can also chelate transition metal ions (mainly iron and copper) catalyzing reactions with ROS generation. The most important element in the structure of coumarins, which determines their ability to capture ROS, is an electron-donating hydroxyl group. Blockade of this group by methoxy or glycoside moiety significantly reduced antioxidative abilities of hydroxyl group [210]. Coumarins containing hydroxyl groups are most effective with substitution at the C6 or C7 positions [211] and the presence of two hydroxyl groups, particularly in the ortho position, significantly increases the reactivity of coumarins. Ortho-dihydroxycoumarins exhibit strong ability to capture superoxide radicals (approximately 4–6 times higher than trolox), but meta derivatives exhibit much less radical scavenging. Adjacent hydroxyl groups at the aromatic ring stabilize the phenoxy radical formed due to the presence of intramolecular hydrogen bonding. If the molecule contains three adjacent hydroxyl groups, stabilizing the nascent radical is even greater and the compound is a stronger antioxidant [212]. The substitution with methyl group at the C-4 position in 4-methylesculetin (6,7-dihydroxy-4-methylcoumarin) (**7**) (Figure 2) causes a significant decrease in reactivity [209]. Coumarins reduce the DPPH radical with a comparable degree as known antioxidants. The antioxidant capacity of esculetin (6,7-dihydroxycoumarin) (**8**) (Figure 2) and 4-methyl-7,8-dihydroxycoumarin (**9**) (Figure 2) in reaction with DPPH radical is comparable to quercetin (**3**) antioxidant capacity (EC_50_ values approximately 25–27 μM) [213]. Antioxidant activity of coumarins against the DPPH radical increases in the presence of an electron donating substituent, such as −OH, −CH_3_, −Cl, −OCH_3_ [214]*,* while it decreases in the presence of a large substituent at the C-3 position due to the steric obstacle [215]. Coumarins also affect the activity of antioxidant enzymes such as superoxide dismutase, catalase, glutathione peroxidase and S-glutathione transferase in the liver, kidney and plasma of rats [216]. Umbelliferone (7-Hydroxycoumarin) (**10**) (Figure 2) and esculetin (**8**) increase the concentration of vitamins E and C, as well as GSH [217]. Esculetin (**8**), fraxetin (7,8-dihydroxy-6-methoxycoumarin) (**11**) (Figure 2), daphnetin (**12**) (Figure 2) are more powerful antioxidants than caffeic acid or vitamin E. However, these coumarins show comparable activity with (+)-catechin (**13**) (Figure 2) [218]. The consequence of the antioxidant activity of coumarins is the decrease in cell components modified by oxidative effects. Coumarins prevent oxidative damage of protein and DNA [219,220] more effectively than their acidic and glycosidic forms [218]. Methyl coumarin derivatives, in contrast to hydroxyl derivatives, can show prooxidative activity, by generation of free radicals and reduction in transition metal ions. It was shown that 4-methylcoumarin (**14**) (Figure 2) and its derivatives can react with peroxide radical and form benzyl and phenoxy radicals, which may initiate lipid peroxidation [221,222]. The oxidizing potential of 7-hydroxy-4-methylcoumarin (**15**) (Figure 2) is significantly higher than for α-tocopherol (**16**) (Figure 2). Phenoxy radical of **15** in the reaction with **16** can give rise to the tocopheryl radical, which also initiates lipid peroxidation [221]. Prooxidative properties of coumarins result from the possibility of reducing Fe(III) and Cu(II) ions, with the formation of Fe(II) and Cu(I) ions, respectively, which catalyze the Fenton reaction. 5,7-Dihydroxy-4-methylcoumarin (**17**) (Figure 2) derivatives oxidize LDL in the reaction catalyzed by copper ions [223]. Inophyllum A (**18**) (Figure 2), which is a natural analogue of coumarin, had very promising results for docking studies with main COVID-19 protease (PDB ID:5N5O). The binding energy of inhibition potential of this enzyme was −8.4 kcal/mol, but additional studies are important for medicinal use [224].

### 5.3. Chromones as Antioxidants

**Figure 3:**
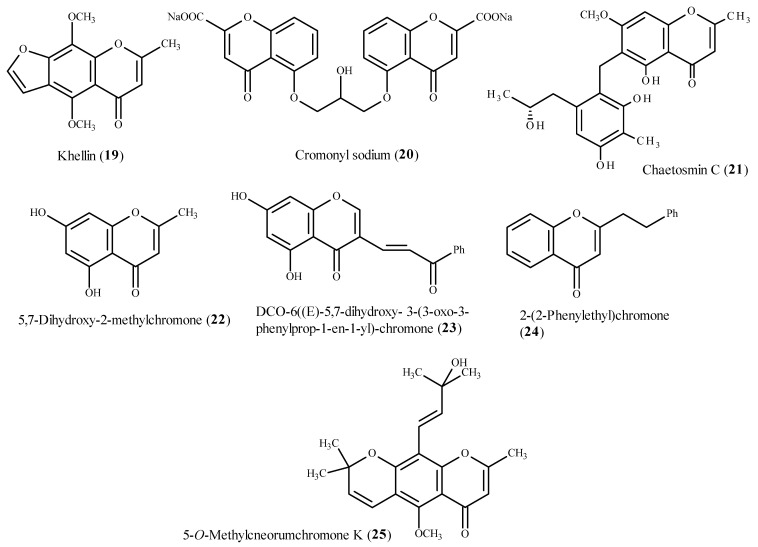
Structures of chromone derivatives mentioned in the Section 5.3.

Chromones are derivatives of 1,4-benzopyrone and are coumarin isomers. They are found in the different botanical parts. Most of them are simple compounds, but they also occur as a glycoside with substitution at C-2, C-5 and C-7 positions. Some of chromones have a substitution at the C-5 position with a long chain and additional ring. Chromones possess anticancer, antibiotic, antioxidant, antimalarial, as well as anti-inflammatory, antibacterial, antispasmolytic, anti-HIV activities [225,226]. Many in vitro and in vivo studies were performed to evaluate pharmacological properties of chromones [227] and also antihypertensive, antiviral, antifungal activity was investigated in the experimental models [228]. Khellin (**19**) (Figure 3) from *Ammi visnaga* (L.) is clinically used to treat *vitilgo* [229] and *angina pectoris* and is allowed to be used in the treatment of asthma. The other compound of chromone derivatives, such as cromolyn sodium (**20**) (Figure 3) is also used in the treatment of asthma and was approved by US Food and Drug Administration (FDA) in 1973.

It was established by Yadav et al. [230], that the presence of double bond, carbonyl group, 3′,4′dihydroxy group in the ring B in addition to the C-3 and C-5 hydroxyl groups results in elevated potential of free radical scavenging. Chaetosemin C (**21**) (Figure 3) from *Chaetomium seminudum* at concentration of 50 µM, scavenge 50.7% of DPPH free radicals [231]. 5,7-Dihydroxy-2-methylchromone (**22**) (Figure 3), displayed strong antioxidant activity in in vitro and ex vivo studies, and hence, was further studied for anti-aging activity [232]. 7,8-Dihydroxy-2-(3′-trifluoromethylphenyl)-3-(3″-trifluoromethylbenzoyl)chromone displayed stronger radical scavenging and metal chelating activities than reference compounds, such as butylated hydroxytoluene, trolox and vitamin E [233]. According to Mazzei et al. [234], chromones substituted with a 1-piperidinyl group can be considered as strong anti-inflammatory compounds, because they inhibited reactive oxygen species production by activated human neutrophils. Sesquiterpene chromones from *Ferula fukanensis* roots suppressed NO production activated by lipopolysaccharide (LPS) and recombined interferon-c in the murine macrophage cell line RAW 264.7 [235]. It was found by Liu et al. [221], that DCO-6((E)-5,7-dihydroxy-3-(3-oxo-3-phenylprop-1-en-1-yl)-chromone (**23**) (Figure 3) inhibited reactive oxygen species (ROS), depended on activation of the TRAF6-ASK1-p38 pathway. Chen et al. [236] suggested that the 2-(2-phenylethyl)chromone (**24**) (Figure 3) derivatives inhibited NO production in RAW 264.7 cells, with IC_50_ values ranging from 5.12 to 22.26 µM. It was reported by Liu et al. [237], that 5,7-dihydroxy-4-oxo-4H-chromen-3-yl)methyl esters inhibited NO production in RAW264.7 cells and the IC_50_ values ranged from 0.35 to 2.20 µM. (6-(3-Methylbut-2-enyl)allopteroxylinmethyl ether, 3,3-dimethylallylspatheliachromene-methyl ether and 5-*O*-methylcneorumchromone K (**25**) (Figure 3) from the roots of *Dictyoloma vandellianum* suppressed NO and cytokine production, stimulated by LPS and IFN-c at concentrations in the range from 5 and 20 µM [238].

## 6. Antioxidant Complexes of Flavonoids, Coumarins, Chromones with Metal Ions

### 6.1. Complexes of Flavonoids with Metal Ions

**Figure 4:**
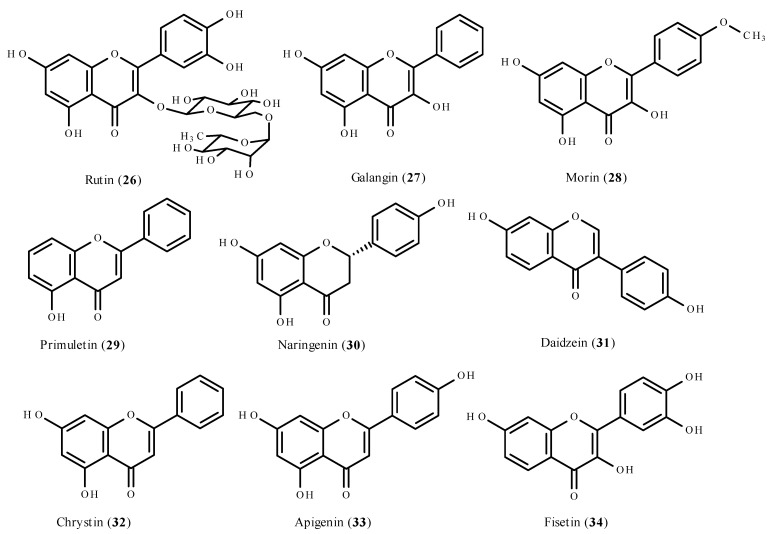
Structures of compounds **26**–**34**.

**Figure 5:**
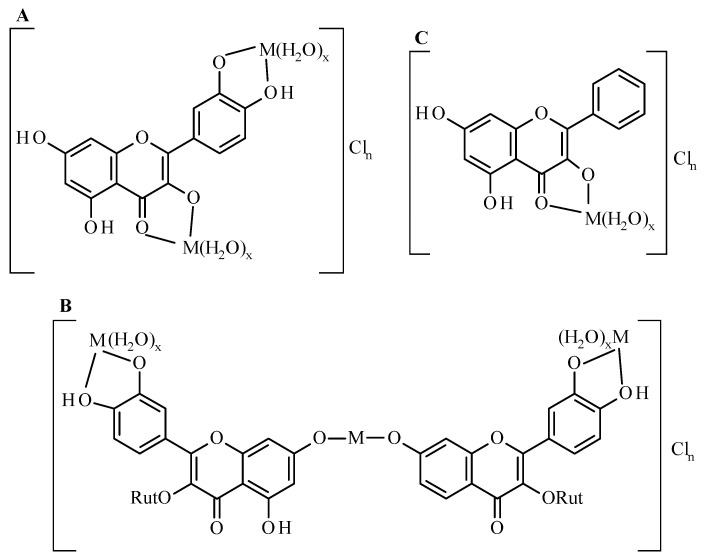
Proposed structures of Zn(II) and Al(III) complexes with quercetin (**A**) M are Al(III) and Zn(II), x = n = 4 for Al(III), x = n = 2 for Zn(II); rutin (**B**) M are Al(III) and Zn(II), x = 4, n = 5 for Al(III), x = n = 2 for Zn(II); galangin (**C**) x = 4 and n = 2 for Al(III), x = 2 and n = 1 for Zn(II).

**Scheme 1:**
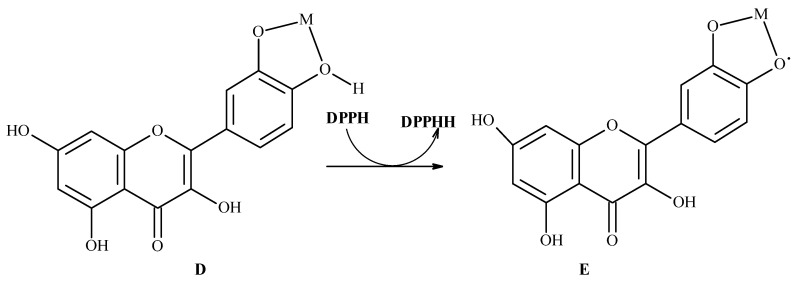
Scheme of oxidation pathway for metal-quercetin complex through DPPH radical and with formation of semiquinone radical intermediate [241].

Antioxidant properties of flavonoids are based on radical scavenging, but mainly on chelating properties [239,240]. Antioxidant properties of quercetin (**3**), rutin (**26**) (Figure 4), (+)-catechin (**13**), galangin (**27**) (Figure 4) with transition metals such as Al(III), Zn(II) (Figure 5), Cu(II), Fe(II), were investigated. The analysis was performed with use of diphenyl-2-picrylhydrazyl (DPPH). DPPH is a non-enzymatic method and allows the samples to be evaluated through their scavenging activity of DPPH radical by donation of one electron or hydrogen. As a result, a semiquinone complex was obtained, which is stabilized by conjugation with 3′-OH substituent and also by the metallic center (Scheme 1) [241].

In general, the antioxidant activity of these complexes was higher than free flavonoids mentioned before. That can be explained by the presence of superoxide-dismutating center, which can be an additional radical scavenging metal center [241,242,243]. Antioxidant efficiency (AE) values characterize the strength of antioxidant activity according to the scheme:

AE < 1·× 10^4^—low; 1 × 10^4^ < AE < 5 × 10^4^—medium; 5 × 10^4^ < AE ≤ 10·× 10^4^—high; AE > 10·× 10^4^—very high.

The formula for AE is expressed as follows:

AE = (1/EC_50_) × TEC_50_,

where EC_50_ is an amount of antioxidant needed to reduce by 50% and TEC_50_ is a time needed to reach the EC_50_ concentration. The results of TEC_50_ and AE values, scavenging ratio of DPPH and antioxidant activities classification are given in Table 1. EC_50_ values depend on differences between semiquinone complexes and the ground state energies of complexes. Stabilization of A leads to increase in ΔE and decrease in antioxidant activity. For case B, this relationship is reversed.

Considering this selection, it should be concluded that complexes of quercetin are antioxidants with the highest activity. Al(III) complex of quercetin (**3**) is a very active antioxidant agent and that can be explained by stabilization effect of this ion on radicals from flavonoid phenoxyl. Moreover, Al(III) possesses empty *p* orbitals which can be good acceptors of π electrons [244].

**Figure 6:**
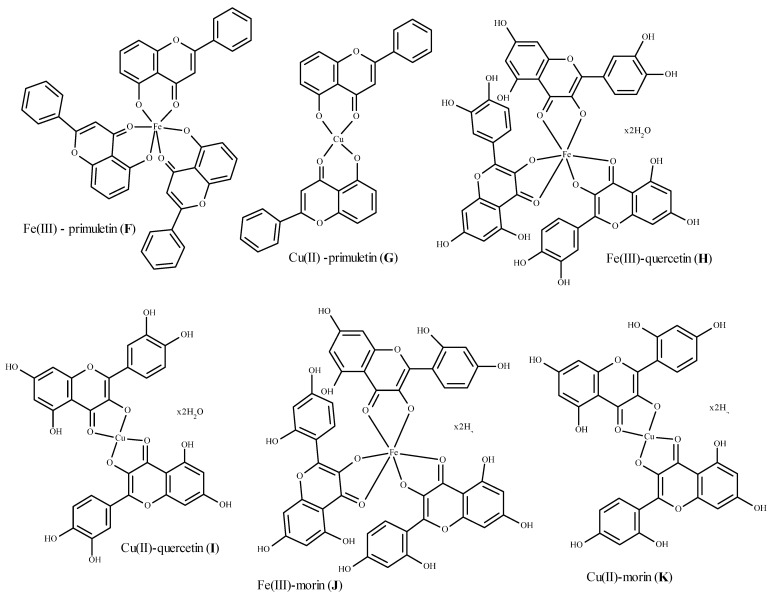
Structures of Fe(III) and Cu(II) complexes (**F**–**K)** with primuletin (**29**), quercetin (**3**) and morin (**28**).

It was shown that Fe(III) complex with primuletin (5-hydroxyflavone) (**29**) (Figure 4) decreased ROS activity and elevated catalase (CAT) and superoxide dismutase (SOD) scavenging activity in the control group of rats and rats pretreated with alloxan. Alloxan causes diabetes in laboratory animals through damaging β-cells in the liver. It should be noticed that ROS level is elevated in the diabetes. The complexes of Cu(II) and Fe(III) with flavonoids such as morin (**28**) (Figure 4), quercetin (**3**)**,** primuletin (**29**) were studied by use of UV-Vis spectroscopy and cyclic-voltammetry (Figure 6). It was revealed that these complexes possess better antioxidant activity than ligands. It was also found that through hydrogen atom transfer, complexes deactivate oxidants. Primuletin (**29**) complexed by Fe(III) showed antioxidant potential through direct scavenging of free radicals and increased activity of CAT and SOD [245].

Free radical scavenging activity of Ce(IV) complexes with naringenin (**30**), daidzein (**31**), chrysin (**32**) (Figure 4) was evaluated with use of DPPH radical scavenging assay (RSA). In the different mixtures of DMSO and water, the antioxidant activity of flavonoids in complex with Ce(IV) ions increased. It was also observed that flavonoid with the same number of hydroxyl substituent, but from different subclasses, have comparable values of RSA. This can be demonstrated for chrysin (flavone) and daidzein (isoflavone). The order of metal chelation is following: daidzein > chrysin > naringenin [246].

The antioxidant properties of Pb(II) complexes with quercetin (**3**), kaempferol (**4**), chrysin (**32**), apigenin (**33**) (Figure 4) were checked with use of FRAP and ABTS assays. Ferric Reducing Power Assay (FRAP), is a method for determination of reducing power of samples. There is used Fe(III)-TPYZ complex, which is reduced to Fe(II)-TPTZ complex in the acidic environment. The Fe(II)-TPTZ complex is blue and absorbs light at 593 nm and that allows evaluation of antioxidant properties of samples. ABTS is an analysis for measuring radical scavenging capacities and use a blue colored nitrogenous radical, which absorbs light at 734 nm. The reaction with antioxidant leads to change in blue color of ABTS. That analyses showed that the best radical scavenging activity had quercetin (**3**) and kaempferol (**4**). Almost all complexes of flavonoids, except quercetin (**3**) were obtained in a 2:1 ratio. According to the results, quercetin (**3**) and kaempferol (**4**) easier react with Pb(II) ions as well as show stronger antioxidant properties, than chrysin (**32**) and apigenin (**33**) [247].

**Figure 7:**
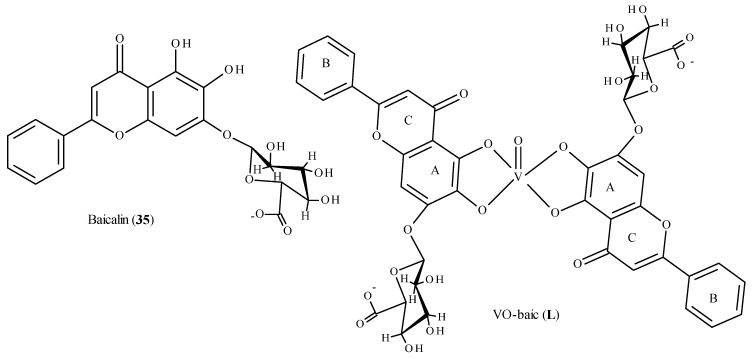
Structures of baicalin (**35**) and its vanadium(V) complex VO(baic) (**L**).

Baicalin (**35**) (Figure 7) is a main compound of *Scutellariae radix* and shows antioxidant, antiallergic and anti-inflammatory actions. It was reported that complexation of baicalin (**35**) by oxovanadium(IV) (Figure 7) improved antioxidant properties of this ligand and that was supported by results of antioxidant tests. Nitrobluetetrazolium (NBT) assay was used to examine activity of superoxide dismutase (SOD) and the VO(baic) (**L**) (Figure 7) ability to inhibit reduction in NBT by superoxide anion was checked. A complex of **35** was also used to scavenge 1,1-diphenyl-2-picrylhydrazyl (DPPH) radicals and the measurement was made according to modified method of Yamaguchi et al. [248]. Besides, 2,2-azobis(2-amidinopropane)dihydrochloride (AAPH) [249] was used in the experiment, because it generated free radicals during thermal decomposition and inhibition of peroxyl radicals of **35** was evaluated [250].

**Scheme 2:**
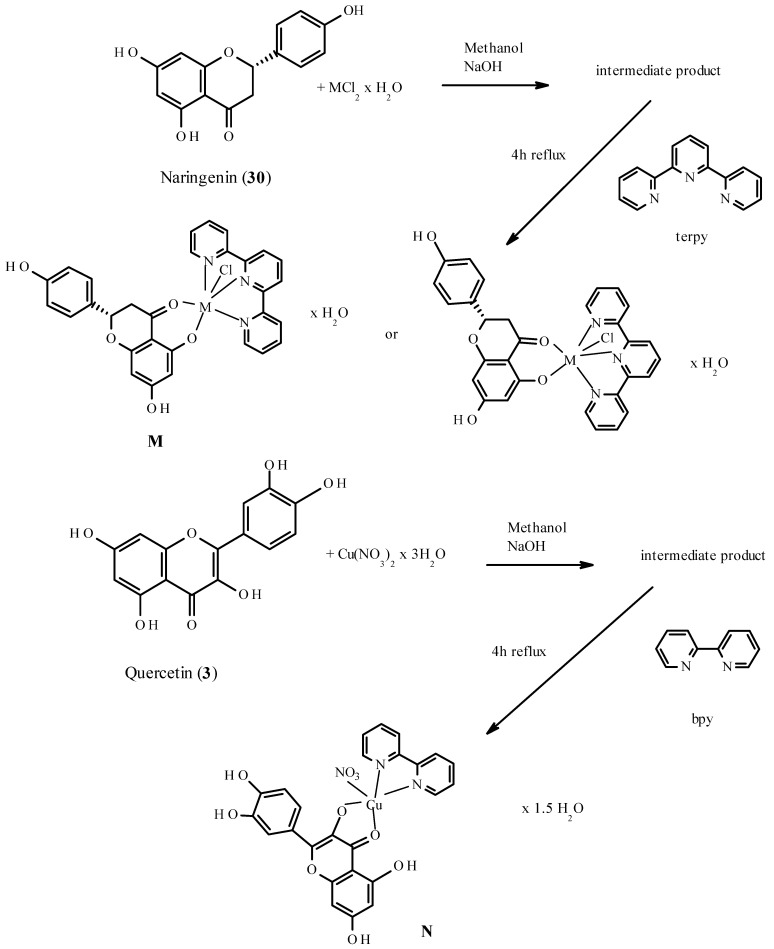
Synthesis and possible structures of Ni(II) and Cu(II) complexes with quercetin (**3**) and naringenin (**30**).

Complexes of quercetin (**3**) and naringerin (**30**) with nickel(II) and copper(II) (Scheme 2) were compared in terms of their antioxidant capacities and phenolic contents. The phenolic contents of these complexes were, respectively 0.17 ± 0.01; 0.21 ± 0.01; 0.67 ± 0.04 mg GAE/kg and antioxidant capacities was 0.0020 ± 0.001; 0.030 ± 0.001; 0.720 ± 0.001 mg TE/kg. It is important that number of hydroxyl groups correlates with antioxidant activity of flavonoid. Additionally, E_pa_ value determines antioxidant activity of flavonoid, because if the E_pa_ is lower, the antioxidant activity of flavonoid is higher. Quercetin (**3**) (E_pa_ = 0.33 V) is a better antioxidant compound than naringenin (**30**) (E_pa_ = 0.45 V) and that influences antioxidant properties of their complexes, because quercetin (**3**) complexes have stronger antioxidant properties than naringenin (**30**) complexes [251].

Chelating activity test of Fe(II) ions, total antioxidant activity test with use of ferric thiocyanate and thiobarbituric acid, total reductive capability with use of potassium ferric reduction were the analyses that were applied for determination of antioxidant activity of chromone derivatives. Flavonoids inhibit the Fenton and Haber-Weiss reactions, because of good chelation of metals. Moreover, flavonoids retain radical scavenging capacity after complexing with metal ions [252]. Flavonoid compounds with a catechol substitution at C-2, such as luteolin (**1**), quercetin (**3**), fisetin (**34**) (Figure 4), were effective antiradicals [253] in the DPPH test. That may be caused by the presence of more than two hydroxyl substituents and the presence of two complexation active site. Compounds **36**–**41** (Table 2) exhibited good antioxidant activity with use of chelating activity test of Fe(II) ions, DPPH and FTC tests (Table S1 in Supplementary Materials). The ferrous ion (Fe(II)) chelating method was used for determination of metal chelating activity of chromone derivatives. The color complex of Fe(II)-ferrozine which is red, is changed in the presence of chelating agents. This change in color is used for estimating the chelating activity of compounds. Ferric thiocyanate method (FTC) is used for evaluation of the peroxide amount, which may be released during lipid peroxidation. During the peroxidation of lipids, the ferric ions are formed as a result of reaction peroxide with ferrous chloride. The ferric ions react with ammonium thiocyanate and produce a red product. The absorbance is higher if the color of product is darker. Thiobarbituric acid method is an assay based on the reaction of malonodialdehyde, which is a product of lipid peroxidation with thiobarbituric acid. That leads to formation of color complex and its absorbance is measured at 532 nm [233].

**Figure 8:**
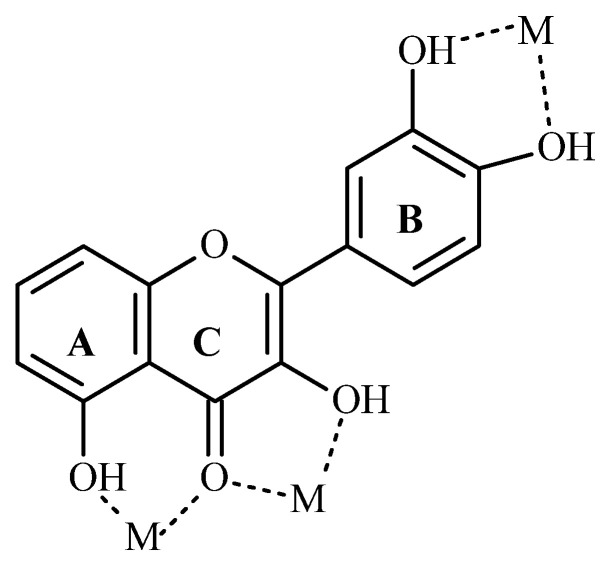
General structure of flavonoids with indicated sites of binding with metal ions.

The antioxidant capacity was determined by CRAC assay, which is an electrochemical assay for measuring antioxidant capacity. In this method, cerium (IV)sulfate is used, which is an oxidant. During the analysis a decrease in concentration Ce (IV) ions was observed after 4 min reaction with antioxidant, beginning with a potential E_1_ approximately 1.29 V for 2 seconds and reducing to the potential E_2_ = 0.8 V for 10 seconds. Quercetin (**3**), morin (**28**) and fisetin (**34**) showed an increase in their antioxidant capacity around 32%, 15% and 28% (Table S2), mainly by presence of active sites, which are involved in the interaction with metals. The complexes of Fe(II) were formed by binding with hydroxyl substituent at C3 and carbonyl group at C4 in the C ring (Figure 8). Catechin (**13**) and chrysin (**32**) did not show significant antioxidant capacity because of unfavorable site of binding with metal ions [254].

Complexes of Cu(II), Fe(II), Fe(III), Ni(II), Zn(II) with rutin (**26**) are better anti-inflammatory agents [255,256] and a complex of Mg(II) with rutin (**26**) is active against rat paw edema induced by serotonin. Fe(III) complex of rutin (**26**) suppresses lung edema [242], but it stimulated release of oxygen radicals from macrophages. Other studies indicated that complexes of Cr(III) [257], Cu(II) [258], Fe(II), Zn(II) [239], VO(IV) are better radical scavengers than ligands.

### 6.2. Complexes of Coumarins with Metal Ions

**Scheme 3:**
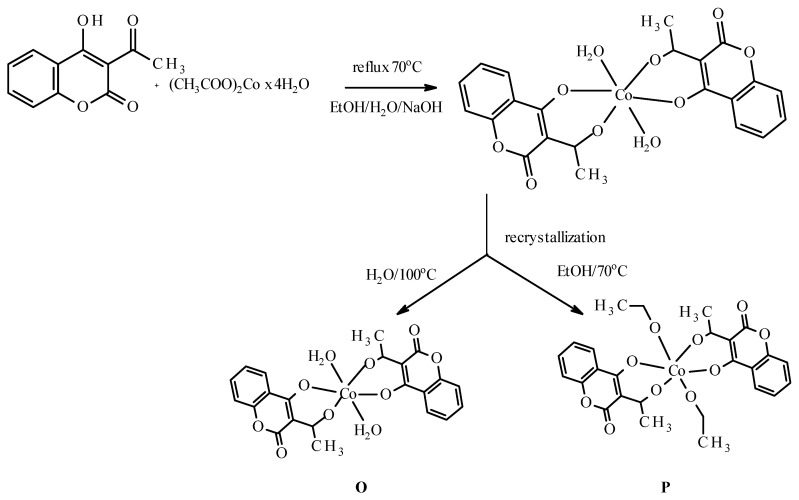
Synthesis and recrystallization of Co(II) complex with 3-acetyl-4-hydroxy-2*H*-chromene-2-one **O** and **P**.

The antioxidant activity is related to prevention of development of neoplasms caused by chemical agents in animals. Coumarins can restrain formation of DNA adducts with polycyclic aromatic hydrocarbons (PAH) in mouse tissues [259,260,261,262]. Two new Co(II) complexes **O** and **P** (Scheme 3) possess antioxidant potency stronger than their ligand. The comparison of the free radical scavenging activity of these newly synthesized products with ascorbic acid IC_50_ = 21 µmol/L (3.75 mg/L) [214] showed that these coumarin complexes are powerful antioxidants and can be considered as competitors in the pharmaceutical field [263].

**Scheme 4:**
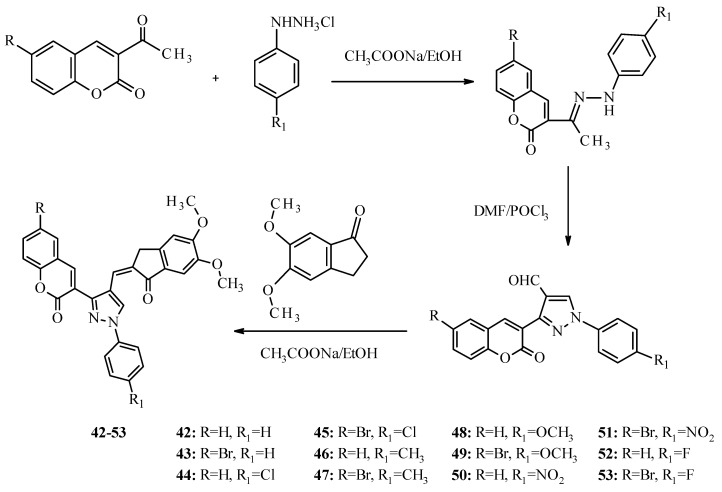
Synthesis of coumarin derivatives **42**–**53**.

The coumarins from **42** to **53** (Scheme 4) in the test with DPPH showed considerable radical scavenging activity, but the strongest antiradical activity had compounds from **46** to **49** with IC_50_ values from 54.14 to 59.76 µg/mL. The IC_50 of_ compound **42** was 64.75 µg/mL and compounds **44** and **52** with halogen substituents showed good scavenging effect with IC_50_ 70.14 µg/mL and 88.29 µg/mL, respectively (Table S3). The IC_50_ value of reference standard BHT was 46.95 µg/mL. The chelating effects on ferrous ions was measured and it was established that, compounds containing functional groups such as −OH, −SH, −COOH, C, O, −NR_2_, oxygen show activity of metal chelation [260]. Compounds with substitution of electron donating groups on the indenone and phenyl ring **46**–**49** exhibited excellent activity with IC_50_ value in the range 53.75–62.30 µg/mL. Moreover, compounds **42** and **52** displayed satisfactory activity with IC_50_ 65.95 µg/mL and 7.76 µg/mL. EDTA was a reference compound and its IC_50_ value was 44.11 µg/mL. Total reductive capability is a measurement of potential for reduction Fe(III) ions and is an indicator of electron donating group. The amount of Fe(II) complexes was measured as the formation of Perl’s Prussian blue at 700 nm. If the absorbance increased at 700 nm, the reducing ability of compounds increased [264]. Compound **48**, which is substituted with two methoxy groups on indenone ring and substitution with methoxy group on phenyl ring had the best reducing power with IC_50_ = 58.01 µg/mL. The IC_50_ value of butylated hydroxyanisole (BHA), which was a reference compound had IC_50_ = 50.00 µg/mL [265].

**Scheme 5:**
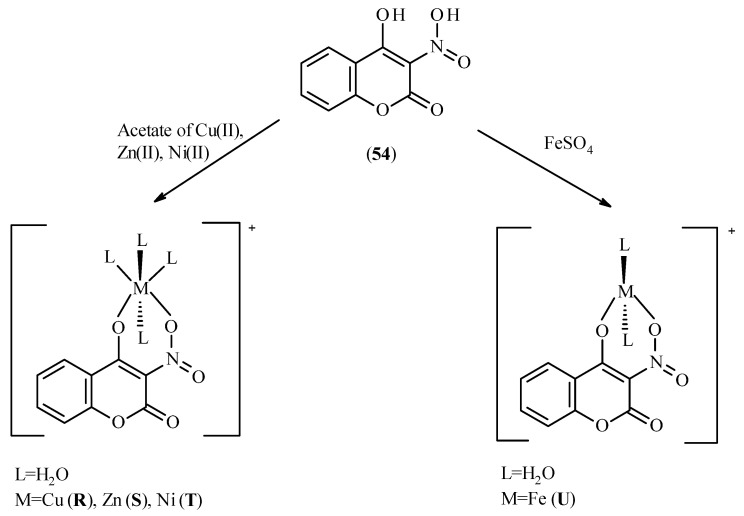
Possible structures of Cu(II) (**R**), Zn(II) (**S**), Ni(II) (**T**) and Fe(II) (**U**) complexes with 4-hydroxy-3-nitrocoumarin (**54**).

4-Hydroxy-3-nitrocoumarin (4H_3_NC) (**54**) and its complexes with Cu(II), Zn(II), Ni(II), Fe(II) ions (Scheme 5) were tested with use of an indirect enzymatic method described in Sadler et al. [266]. The antioxidant activity was measured by the absorbance decrease as a result of competition with NBT (nitrobluetetrazolium). NBT, which reduces formazan, accept electrons, which are donated by superoxide radical. The percentage inhibition for 4H_3_NCCu was 91.2% and it was higher than for 4H_3_NC (63.7%). Complexes 4H_3_NCNi and 4H_3_NCFe did not show antioxidant activity (Table S4) [267].

### 6.3. Complexes of Chromones with Metal Ions

**Figure 9:**
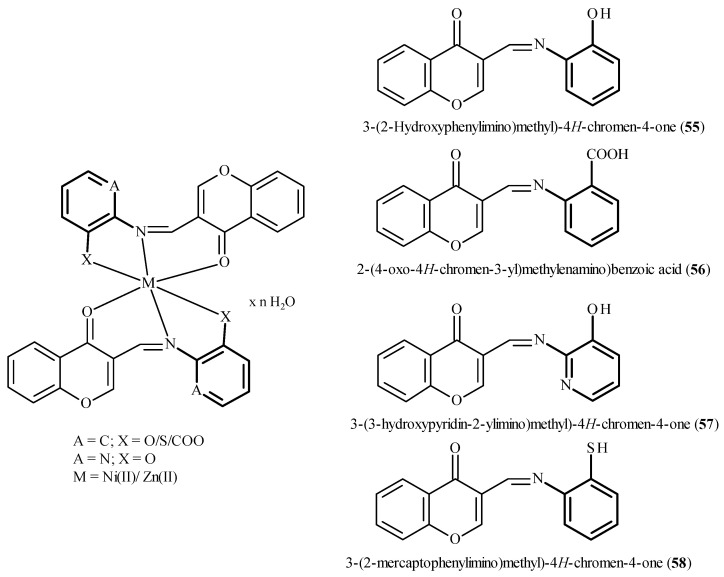
Structures of compounds **55**–**58** (Schiff base ligands) and predicted structure of their complexes with Ni(II) and Zn(II) ions.

The Ni(II) and Zn(II) complexes of chromone derivatives (**55**–**58**) (Figure 9) were analyzed with DPPH free radical scavenging method. The ability of the complexes to donate electrons to DPPH was monitored as changes in the absorption at 517 nm with use of UV-VIS spectrophotometer. Complex [Zn (**58**)_2_]⋅H_2_O (IC_50_ = 0.69 µg/mL) exhibited comparable antioxidant activity to BHT (butylatedhydroxyltoluene) (IC_50_ = 0.67 µg/mL), which was a standard drug. The complexes [Ni (**55**)_2_], [Ni (**58**)_2_], and [Zn (**58**)_2_]⋅H_2_O showed moderate antioxidant activity in comparison to the rest of complexes, antioxidant activity of which was weak [268].

## 7. Binding Motifs and Supramolecular Architecture for Crystal Structure of Plant Compounds and Their Metal Complexes

**Figure 10:**
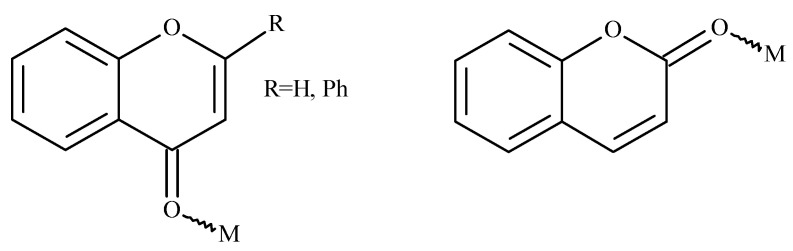
The query used in the search of the database.

For the discussion concerning the structural features of flavonoids, chromones and coumarin as complexes the crystallographic database was search according to Figure 10, where M = Zn(II), Cu(II), Fe(III), Ni(II) and Al (III). Among 104 different metal complexes, 60 flavonoids complexes, 20 chromones complexes and 24 coumarins complexes were found.

### 7.1. Flavonoids (Flav)–Metal Complexes

**Figure 11:**
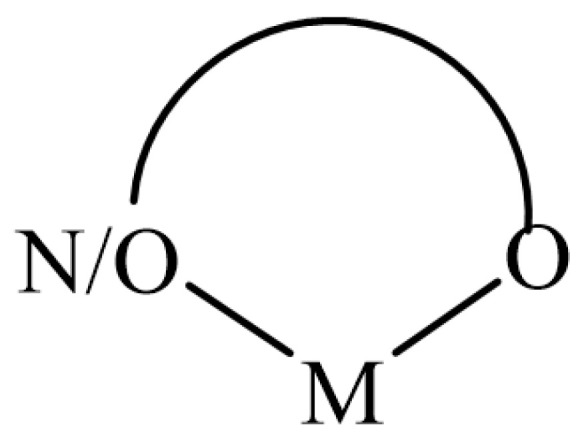
Coordination modes for the flavonoids complexes.

In so far reported X-ray structures of metal-flavonoids complexes, the flavonoid is bound to the metal in two different binding modes shown in Figure 11, either in a bidentate chelating mode via two oxygens (3-hydroxy, 4-carbonyl) or in a bidentate mode via oxygen (4-carbonyl) and nitrogen (3-amino).

**Figure 12:**
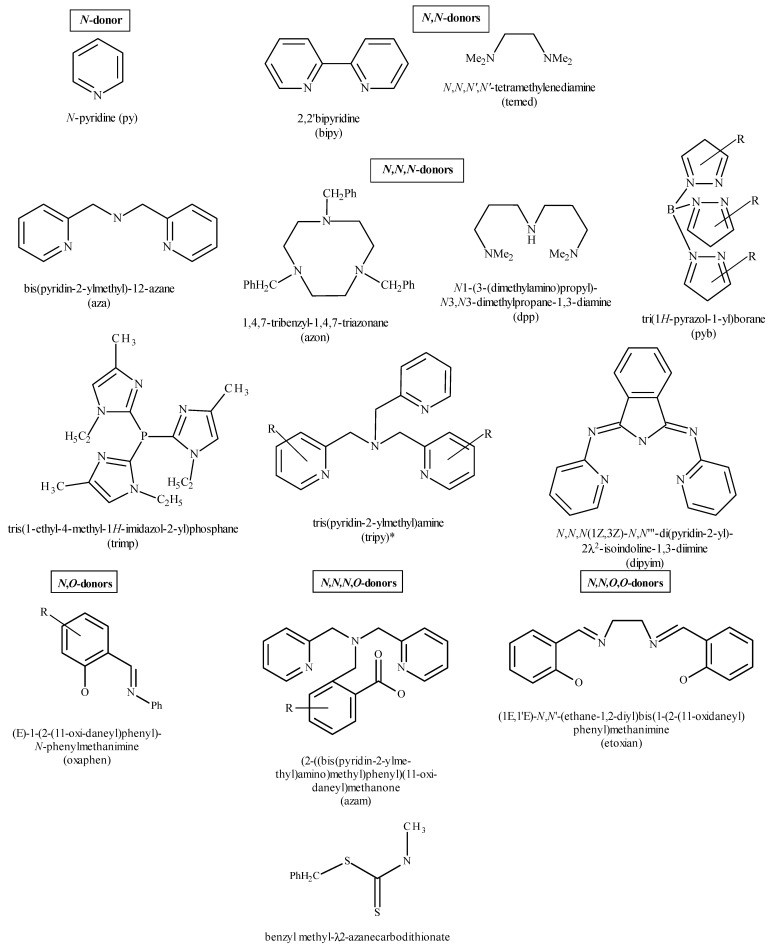
Type of ligands in flavonoid complexes. Asterix “*” indicates that ligand tripy could be in tridentate or tetradentate mode.

Among all the complexes, there are only a few bis complexes: [Cu(Flav-*O*,*O*)_2_(py-*N*,*N*)_2_] [269], [Cu(Flav-*O*,*O*)_2_] [270] and [Fe(Flav-*O*,*O*)_2_Cl,CH_3_OH] [271]–with additional Cl and CH_3_OH ligands [Ni(Flav-*O*,*O*)_2_(py-*N*)_2_] [272] with pyridine ligands and one tris complex [Fe(Flav-*O*,*O*)_3_] [273]. Other co-ligands bound to M may be divided into 10 categories:

- *O*-donors such as H_2_O, ClO_4_, NO_3_, dimethylsulfoxide (DMSO), 2-(2-hydroxyphenyl)-2-oxoacetic acid;
- *P*-donors such as PPh_3_;
- *F*-donors such as BF_4_;
- *N*-donors including pyridine(py);
- *N*,*N*-donors ((2,2′bipyridine (bipy), *N*,*N*,*N*′,*N*′-tetramethylenediamine (temed));
- *N*,*N*,*N*-donor (bis(pyridin-2-ylmethyl)-l2-azane (aza), 1,4,7-tribenzyl-1,4,7-triazonane (azon), N1-(3-(dimethylamino)propyl)-N3,N3-dimethylpropane-1,3-diamine (dpp), tri(1*H*-pyrazol-1-yl)borane (pyb), tris(1-ethyl-4-methyl-1*H*-imidazol-2-yl)phosphane (trim), tris(pyridin-2-ylmethyl)amine (tripy), *N*,*N*,*N* (1*Z*,3*Z*)-*N*,*N*′′′-di(pyridin-2-yl)-2l2-isoindoline-1,3-diimine (dipyim));
- *N*,*N*,*N*,*N*,-donor tris(pyridin-2-ylmethyl)amine (tripy);
- *N*,*O*-donors:(E)-1-(2-(l1-oxidaneyl)phenyl)-*N*-phenylmethanimine (oxphen);
- *N*,*N*,*N*,*O*-donor (2-((bis(pyridin-2-ylmethyl)amino)methyl)phenyl)(l1-oxidaneyl)methanone (azam);
- *N*,*N*,*O*,*O*,-donor (1E,1′E)-*N*,*N*′-(ethane-1,2-diyl)bis(1-(2-(l1-oxidaneyl)phenyl)methanimine) (etoxian) (Figure 12).

#### 7.1.1. Cu(II)-Flav Complexes

The crystal structure of four Cu-flavonoid complexes: [Cu(Flav-*O*,*O*)(oxphen-*N*,*O*)] [274], [Cu(Flav-*O*,*O*)(oxphen-*N*,*O*)] [274] and [Cu(Flav-*O*,*O*)(bipy-*N*,*N*)] [275] and [Cu(Flav-*O*,*O*)_2_] [276] are coordinated in the bidentate chelating mode by *O*,*O* atoms from the flavonoid part and *N*, *N*/*O* from the co-ligands. Four of the Cu(II) complexes are four-coordinated, with a slightly distorted square planar geometry, while the complex [Cu(Flav-*O*,*O*)(PPh_3_)_2_] [276] has tetrahedral geometry with (Flav-*O*,*O*) ligand coordinated in bidentate mode and two monodentate PPh_3_.

Five-coordinated Cu(II)-Flav complexes are in three different coordination modes. Complexes: [Cu(Flav-*O*,*O*)(azon-*N*,*N*,*N*)] [277], [Cu(Flav-*O*,*O*)(trimp-*N*,*N*,*N*)] [278], [Cu(Flav-*O*,*O*)(tripy-*N*,*N*,*N*)] [279] has distorted square pyramidal geometry (τ in range 0.09 to 0.17), while [Cu(Flav-*O*,*O*)(dpp-*N*,*N*,*N*)] [280] and [Cu(Flav-*O*,*O*)(dipyim-*N*,*N*,*N*)] [281] are in trigonal pyramidal geometry with τ in a range 0.51 to 0.60. General formula for the complexes mentioned are: [Cu(Flav-*O*,*O*)(donor-*N*,*N*,*N*)]. Two complexes with general formula [Cu(Flav-*O*,*O*)(donor-*N*,*N*)(donor-*O*) adopt square pyramidal geometry with τ in a range 0.01 to 0.10. In [Cu(Flav-*O*,*O*)(bipy-*N*,*N*)ClO_4_] [282] flavonoid and 2,2′-bipyridine ligand are bidentate and the other ClO_4_^−^ is monodentate, similarly to [Cu(Flav-*O*,*O*)(bipy-*N*,*N*)acid] [283]. In [Cu(Flav-*O*,*O*)(H_2_O)_3_] [284] oxygen atoms from waters molecule are in monodentate mode with square pyramidal geometry (τ = 0.21).

In the six-coordinated Cu(II) complexes, copper(II) atoms have a distorted octahedral geometry and four different coordination spheres have been found. In centrosymmetric complexes: [Cu(Flav-*O*,*N*)_2_(CLO_4_)_2_] [285], [Cu(Flav-*O*,*N*)_2_(NO_3_)_2_] [286], and [Cu(Flav-*O*,*O*)_2_(BF_4_)]_2_ [287] the two flavonoids molecules are bound via oxygen and nitrogen in bidentate mode, while apical sides are occupied by monodentate perchlorate/tetrafluoroborate/nitrate ions. Another octahedral centrosymmetric complex is [Cu(Flav-*O*,*O*)_2_(py-*N*,*N*)_2_] [269] with two bidentate chelating flav-ligands and two pyridine monodentate ligands. Complex [Cu(Flav-*O*,*O*)(azam-*N*,*N*,*N*,*O*)] [288] is coordinating bidentate flavonoid molecule and azam-ligand in tetrandentate mode.

#### 7.1.2. Zn(II)-Flav Complexes

The coexistence of diverse flavonolate donors and *N*-containing ligands has resulted in the Zn(II)-Flav complexes, in a variety of structures concerning nuclearity and the coordination sphere. In five-coordinated complexes, there are [Zn(Flav-*O*,*O*)(aza-*N*,*N*,*N*)]_2_NO_3_^−^ [289] dinuclear complexes bridging via nitrogen from the aza-donor. The geometry around Zn(II) is trigonal bipyramidal (τ = 0.77). The three complexes [Zn(metoxy-Flav-*O*,*O*)(dpp-*N*,*N*,*N*)]ClO_4_^−^ [290], [Zn(Flav-*O*,*O*)(dpp-*N*,*N*,*N*)]ClO_4_^−^ [291] and [Zn(Flav-O,O)(pyb-*N*,*N*,*N*)] [287] have a similar coordination sphere, ZnO_2_N_3_. The geometry for the complexes mentioned is trigonal bipyramidal with τ = 0.72, 0.71 and square pyramidal with τ = 0.25 for [290,291,292], respectively. The Flav-*O*,*O* ligand is in bidentate and *N*,*N*,*N*-donors in tridentate chelating mode. Another five-coordinated complex, [Zn(Flav-*O*,*N*)_2_(BF_4_)] [286], has different a coordination mode via oxygen and nitrogen bound bidentately and monodentately bound tetrafluoroborate ion (τ = 0.03-SQP geometry).

In the six-coordinated Zn(II) complexes, zinc(II) atoms have a distorted octahedral geometry, while a different coordination sphere was found. In complexes [Zn(Flav-*O*,*O*)(tripy-*N*,*N*,*N*,*N*)]ClO_4_^−^CCl_3_ [293] and [Zn(Flav-*O*,*O*)(tripy-*N*,*N*,*N*,*N*)]ClO_4_^−^CH_3_CN the coordination sphere is the same, flavonolate donors are bidentate and tripy-ligand tetradentate. Complex [Zn(Flav-*O*,*O*)_2_(H_2_O)_2_] [294] is centrosymmetric with two Flav-*O*,*O* ligands occupying equatorial plane and two oxygens in apical positions. In flavonoid moiety the sulfonato group coordinated sodium cation is substituted. In consequence the supramolecular 3-D structure is observed in the crystal lattice. The coordination sphere in the complex [Zn(Flav-*O*,*O*)(H_2_O)2(SO_3_)_2_] [294] is completed by bidentate Flav-*O*,*O* ligand with two waters in equatorial positions and two sulfonyl oxygen atoms (from adjacent complex) in axial positions, which leads to assembling the 1D-ladder-like chain coordination polymer. Complex [Zn(Flav-*O*,*O*)(bipy-*N*,*N*)(ClO_4_)_2_] [295] is dinuclear bridging via nitrogens and oxygens with weakly bound perchlorate ions. Complex [Zn(Flav-*O*,*O*)_2_(temed-*N*,*N*)] [296] has distorted octahedral geometry with bidentate mode of ligands.

#### 7.1.3. Co(II)-Flav Complexes

In the Co(II)-Flav complexes, one can distinguish a group of five complexes of general formula [Co(Flav-*O*,*O*)(azam-*N*,*N*,*N*,*O*)] [297,298,299] with distorted octahedral geometry formed by bidentate Flav-*O*,*O* ligand and tetradentate *N*,*N*,*N*,*O*-azam ligand. Other complexes with the same geometries are: [Co(Flav-*O*,*O*)(etoxian-*N*,*N*,*O*,*O*)] [300] and [Co(Flav-*O*,*O*)(tripy-*N*,*N*,*N*,*N*)] [283], where there are different tetradentate ligands coordinated by two nitrogen and four nitrogens, respectively. It is worth mentioning that tripy-ligand is linked here by four *N*-atoms in contrast to the [279] structure. One penta-coordinated Ni(II) complex was found [Co(Flav-*O*,*O*)(pyb-*N*,*N*,*N*)] [292] with slightly distorted square pyramidal geometry (τ = 0.18).

#### 7.1.4. Fe(II/III)-Flav Complexes

In six Fe(II/III)-Flav complexes, six different coordination modes have been observed. Nearly all the Fe(II/III)-Flav complexes are mononuclear (excluding [279] with distorted octahedral geometry with one exception: [292]. Six coordinated complexes [Fe(Flav-*O*,*O*)(azam-*N*,*N*,*N*,*O*)] [297], [Fe(Flav-*O*,*O*)(etoxian-*N*,*N*,*O*,*O*)] [301] differ by atoms which are coordinated in tetradentate mode: *N*,*N*,*N*,*O* vs. *N*,*N*,*O*,*O* from azam and etoxian co-ligand, respectively. Complex [Fe(Flav-*O*,*O*)_3_] [273] is bearing only *O*,*O*-donors as co-ligands in bidentate chelating mode. Its structure is similar to primuletin. Complex [Fe(Flav-*O*,*O*)_2_Cl,CH_3_OH] [271] adopts two Flav-*O*,*O* bidentate donors: chlorine and methanol. Flavanoids ligands are nearly perpendicular, therefore apical sides are occupied by oxygen atom of Flav co-ligand and chlorine atom. The last six-coordinated complex of [Fe(Flav-*O*,*O*)(tripy-*N*,*N*,*N*)0.5O]_2_CH_3_CN,ClO_4_^−^ [279] is dinuclear. Each Fe(III) center has Flav-*O*,*O* ligand and tripy-*N*,*N*,*N* co-ligand and the µ-oxo bridge. Finally, the five coordinated complex [Fe(Flav-*O*,*O*)(pyb-*N*,*N*,*N*)] [290] is distorted square pyramidal with τ = 0.23 bearing *O*,*O* and *N*,*N*,*N* bidentate and tridentate ligands, respectively.

#### 7.1.5. Ni(II)-Flav Complexes

Ni(II) complexes have been categorized on the nature of co-ligands, coordination modes, and geometry around Ni(II). Complexes [Ni(Flav-*O*,*O*)(azam-*N*,*N*,*N*,*O*)] [297] and [Ni(Flav-*O*,*O*)(azam-*N*,*N*,*N*,*O*)] [302] have the same six-coordinated octahedral geometry; however, in [303], the water molecule is as a solvent in the crystal lattice. Similar co-ligands are in complexes of general formula [Ni(Flav-*O*,*O*)(azam-*N*,*N*,*N*,*O*)] [302] where in [303] there is Br-azam co-ligand, while in [299] NO_2_-azam co-ligand with two water molecules beyond the coordination sphere. Other six-coordinated complexes: [Ni(Flav-*O*,*O*)(tripy-*N*,*N*,*N*,*N*)] [279], [Ni(Flav-*O*,*O*)(H_2_O)_4_] [303] and [Ni(Flav-*O*,*O*)_2_(py-*N*)_2_] [272] have the following coordination mode: *O*,*O*-bidentate and *N*,*N*,*N*,*N*-tetradnetate in [279] *O*,*O* bidentate and monodentate four H_2_O in [303] and two *O*,*O* bidentate Flav ligand with two monodentate *N*-pyridine ligands in [269]. Complex [Ni(Flav-*O*,*O*)(tripy-*N*,*N*,*N*,] [279] has the same co-ligand as [279]; however, it is five-coordinated and tripy ligand is coordinated by three nitrogens. In the crystal lattice, the perchlorate ions and dichloromethane solvent are observed. Other five-coordinated complexes [Ni(Flav-*O*,*O*)(pyb-*N*,*N*,*N*,] [304] and [Ni(Flav-*O*,*O*)(pyb-*N*,*N*,*N*,] [292] differ with pyb co-ligands, where in [304] there is 3,5-dimethylpyrazolyl while in [292] 3-mesitylpyrazolyl. For five-coordinated Ni(II) complexes, the distorted square pyramidal geometry is observed with τ in the range from 0.2 to 0.43.

#### 7.1.6. Al(III)-Flav Complexes

Only one complex of Al(III) was found [Al(Flav-*O*,*O*)_3_] [305], which is an example of tris–complex in slightly distorted octahedral geometry. Outside the coordination sphere, there are a chloroform molecule and four water molecules.

### 7.2. Chromones (Chrom)–Metal Ion Complexes

**Figure 13:**
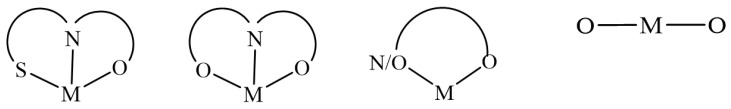
Coordination modes for the chromones complexes.

In the found X-ray structures of metal-chromones complexes, the chromone is bound to the metal in four different binding modes, shown in Figure 13 either in a monodentate, bidentate or tridentate chelating mode via oxygen or nitrogen in case of mono/bidentate mode or via oxygen, nitrogen and sulfur in case of tridentate mode.

The discussion concerning the structural features of M-chromones complexes will be discussed based on the main atom metal. Among all found structures, there are 13 Cu(II) complexes, three Zn(II) complexes and two Ni (II) complexes. Of these, four are dinuclear complexes.

#### 7.2.1. Cu(II)-Chromone Complexes

Most of the Cu(II) chromone complexes bear *O*,*N*,*S* donors as ligands in tridentate mode, where oxygen is bound in chromone moiety in C4 position, while nitrogen and sulphur are a part of thiosemicarbazones and thiosemicarbazides derivatives substituted to the main moiety at position C3. In the dinuclear complexes—[Cu(ThiosemiChrom-O,N,S)NO_3_]_2_ [306], [Cu(ThiosemiChrom-*O*,*N*,*S*)Cl]_2_ [307] and [Cu(ThiosemiChrom-*O*,*N*,*S*)Cl]_2_ [308]—two Cu atoms are double-bridged to a central Cu(II) atom via oxygen (NO_3_) and chlorine atoms. Complex [Cu(Chrom-O)(Chrom-N)Cl]_2_ [309] is also dinuclear, however the ligands are bound to copper atom via oxygen (C4) and nitrogen (C7). Complex [Cu(ThiosemiChrom-*O*,*N*,*S*)SO_4_]_2_ [310] is linked two Cu(II) atoms via one SO_4_^2^-bridge. All mentioned complexes are five-coordinated, with distorted square pyramidal geometry (τ in range 0.06 to 0.33). Other five-coordinated complexes—[Cu(ThiosemiChrom-*O*,*N*,*S*)(H_2_O)(ClO_4_)]ClO_4_^−^ [311], 2[Cu(ThiosemiChrom-*O*,*N*,*S*)(H_2_O)(NO_3_)](NO_3_^−^)_2_ [306], [Cu(ThiosemiChrom-*O*,*N*,*S*)Br_2_] [306]—are mononuclear, bearing thiosemicarbazones and thiosemicarbazides substituents, while one complex [Cu(Chrom-*O*,*O*)(dipyim-*N*,*N*,*N*)] [312] bears bidentate chromon *O*,*O*-ligand and tridentate dipyim-*N*,*N*,*N* co–ligand. For those complexes, one can observe square pyramidal geometry with τ in a range 0.03 to 0.38. Two complexes are six coordinated with slightly distorted octahedral geometry: [Cu(Chrom-*N*,*O*)_2_(ClO_4_)_2_] [313] with two bedentate amino-chroman moiety and two perchlorate ions and complex: [Cu(Chrom-*O*)_2_(ClO_4_)_2_H_2_O] [309] with two monodentate chroman ligands and two waters and perchlorate ions linked to copper(II) atom. One complex with thiosemicarbazone derivative is four-coordinated: [Cu(ThiosemiChrom-*O*,*N*,*S*)Cl] [314] with nearly square planar geometry.

#### 7.2.2. Zn(II)-Chromone Complexes

Two Zn(II) complexes are five-coordinated: [Zn(Chrom-*O*,*N*,*O*)(H_2_O)(NO_3_)] [315] and [Zn(ThiosemiChrom-*O*,*N*,*S*)(H_2_O)(NO_3_)](NO_3_^−^)_2_C_2_H_5_OH [316], with tridentante oxa/thio-chromone ligand and water and nitrate ion coordinated to zinc (II) atom (τ in range 0.08 to 0.22). Complex [Zn(ThiosemiChrom-*O*,*N*,*S*)(Cl)] [314] is in distorted square geometry.

#### 7.2.3. Ni(II)-Chromone Complexes

There are only two nickel(II) complexes: one is [Ni(ThiosemiChrom-*O*,*N*,*S*)(H_2_O)_3_](NO_3_^−^)_2_ [316] bearing bidentate *O*,*N*,*S* thiosemichroman ligand with three water molecules, the second is centrosymmetric with two ThiosemiChrom-*O*,*N*,*S* ligands: [Ni(ThiosemiChrom-*O*,*N*,*S*)_2_]NO_3_^−^ [317]. Both are six-coordinated with slightly distorted octahedral geometry.

### 7.3. Coumarins (Coum)–Metal Complexes

**Figure 14:**
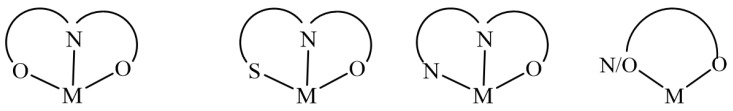
Coordination modes for the coumarins complexes.

Similarly to the previous discussion, the structures of coumarins metal complexes have been categorized according to metal, nature of ligands and coordination number (Figure 14).

#### 7.3.1. Cu(II)-Coumarin Complexes

Among Cu(II) coumarin six-coordinated complexes, there are one dinuclear complex [Cu(Coum*O*,*N*,*N*)_2_]_2_(ClO_4_)_4_,CH_3_NO_2_,H_2_O [318], [Cu(Coum-*O*,*N*,*N*)_2_]_2_(ClO_4_^−^)_2_,CH_3_CN [319], both bearing tridentate Coum-*O*,*N*,*N* ligand, while the latter one is centrosymmetric. Additionally, centrosymmetric is complex [Cu(Coum-*O*,*O*)_2_(H_2_O)_2_ [320]. Five coordinated complexes bear tridentate Coum-*O*,*N*,*O* ligands, its: [Cu(Coum-*O*,*N*,*O*)(C_2_H_5_OH)(NO_3_)] [321] and [Cu(Coumc-*O*,*N*,*O*)_2_(NO_3_)] [322] with τ equals 0.08 and 0.2, what indicate square pyramidal geometry. Four-coordinated complexes: [Cu(Coum-*O*,*N*,*S*)(Cl)] and [Cu(Coum-*O*,*N*,*S*)(Cl)]H_2_O [323] [Cu(Coum-*O*,*N*,*N*)(Cl)] [324] have the ligand in the tridentate mode, all complexes adopt distorted square geometry.

#### 7.3.2. Zn(II)-Coumarin Complexes

Zn(II) complexes with coumarine substituted ligands are mainly six and five-coordinated: [Zn(Coum-*O*,*N*,n)(Cl)](ClO_4_^−^)_2_(CH_2_Cl_2_)_4_ [325], where substituted coumarins ligands are bound by phosphorus atom. Complex [Zn(Coum-*O*,*N*,*O*)(NO_3_)] [326] is polycyclic, while complex [Zn(Coum-*O*,*N*,*O*)] [327] is centrosymmetrical bis-complex with two tridentate ligands as well as [Zn(Coum-*O*,*O*)(H_2_O)] with two bidenate ligands. Five coordinated complexes: [Zn(Coum-*O*,*N*,*O*)(H_2_O)Cl] [328], [Zn(Coum-*O*,*N*,*N*)Cl_2_] [329] have similar tridentate ligands and both have square pyramidal geometry.

#### 7.3.3. Co(II)-Coumarin Complexes

All Co(II) complexes are in octahedral geometry: Three differ by substituent of coumarine molecule; however, the general formula [Co(Coum-*O*,*N*,*O*)(dpp-*N*,*N*,*N*)](ClO_4_)H_2_O [330] and chelating mode is the same. The complex [Co(coum-*O*,*N*,*O*)(CH_3_OH)(NO_3_) [322] is polycyclic.

#### 7.3.4. Ni(II)-Coumarin Complexes

Two Ni(II) complexes were found, both in octahedral geometry, both centrosymmetric bearing tridentate Coum-*O*,*N*,*O* or Coum-*O*,*N*,*N* ligands: [Ni(Coum-*O*,*N*,*O*)]_2_H_2_OOHCNMe_2_ [327] and [Ni(Coum-*O*,*N*)_2_]_2_ [331].

## 8. Application of Plant Compounds and Their Complexes in Medicinal Chemistry and Pharmacy

Plants for medical use have been an important source of pharmaceutical substances since ancient times, and their consumption has increased significantly in recent years. While plant secondary metabolites such as terpenes, alkaloids and cyanogenic glycosides can be used for this purpose, the major pharmacological effect is most often provided by the presence of compounds with a characteristic low-molecular-weight phenolic system such as, i.e., flavonoids, stilbenes or arylbenzofurons [332,333]. Flavonoids are synthesized by plants in response to microbial infection to defense and signaling between plants and microorganisms. As they are one of the largest groups of phytochemicals with proven bioactivity, they show unflagging interest due to their pharmacological activities. It is worth mentioning that most of the flavonoid metal ion complexes are characterized by a similar or even increased therapeutic potential compared to the parent flavonoids [334,335,336].

Flavonoids have the ability to activate human enzyme defense systems and therefore they comprise dietary elements with health-promoting properties. Providing the right amount of flavonoid-rich plants with the diet can contribute to the prevention of many diseases, including cancer, cardiovascular diseases such as atherosclerosis and neurodegenerative diseases such as Alzheimer’s or Parkinson disease [335,336,337]. For example, the prevention of CVD is mainly related to their antioxidant properties. Complex compounds with iron (II) and (III), copper (II) or zinc (II) ions have even greater possibilities of reducing free radical formation and of scavenging free radicals in relation to free flavonoids (e.g., rutin (**26**) or epicatechin). Their action consists, among others, of inhibiting the oxidation of cell membrane lipids and low-density lipoproteins, and increasing the concentration of high-density lipoproteins. As mentioned above, flavonoids have the ability to prevent oxidative stress by interfering with endogenous radical-producing systems and thus protect against cell damage and the development of neurological diseases, diabetes and cancer. Most of the luteolin (**1**) metal ion complexes also exhibit anti-inflammatory properties, which are most likely directly related to their antioxidant activity [338,339,340].

The antimicrobial activity of flavonoids and their metal ion complexes is most likely related to their ability to inhibit the action of bacterial DNA enzymes and protein biosynthesis, damage or disfunction of bacterial cell membranes, and interference with the energy transformations of microorganisms. Likewise, the flavonoids’ antiviral activity is based on the inhibition of various enzymes associated with the life cycle of viruses. Some flavonoids inhibit viral replication by inhibiting the activity of viral RNA polymerase. Such activity was confirmed for sylimarin against hepatitis C virus (HCV) or for quercetin (**3**) against DENV-2. The antiviral activity of flavonoids has been observed and described, inter alia, for other viruses such as human adenoviruses, human cytomegalovirus, HSV-1, HSV-2 and influenza [334,341,342,343].

One of the topics that is currently widely discussed is flavonoids’ potential prophylactic action against SARS-CoV-2 and impact on the treatment of COVID-19 due to their documented antiviral activity against whole group of coronaviruses as a natural ACE2 regulators and inhibitors. ACE2 cellular receptor is an angiotensin-converting enzyme that plays an important role in the transmission of virus to host cells by binding to SARS-CoV spike glycoprotein [335,344,345]. Some flavonoids interact with and inhibit CoV spike glycoprotein. For example, luteolin (**1**) and quercetin (**3**) are able to inhibit the attachment and entry of SARS-CoV virions into human cells after binding to the S2 domain (subunit for the fusion between cellular and viral membranes) of the spike glycoprotein without toxic effect (EC50 of 10.6 µM and EC50 of 83.4 µM, respectively) [337]. In silico studies have also been carried out to identify non-covalent interaction between ligand (inhibitor) and protein (target) of nine flavonoids, including baicalin (**35**), curcumin, galangin (**27**) and epigallocatechin. Molecular docking indicates their high inhibitory potential for the glycoprotein 2019-NCOV (6VSB), especially for the baicalin (**35**) where is even better than in the case of standard drugs such as Abacavir or hydroxychloroquine [346].

Moreover, studies show that in COVID-19 disease, the virus replicates in the epithelial cells of the respiratory tract, which activates the immune system, increases the secretion of a pro-inflammatory cytokine and an intense immune response, leading to damage to lung tissue. This excessive immune response, so called “cytokine storm”, which is manifested by overproduction of pro-inflammatory cytokines such as IL-6, TNF-α and IL-1β, is one of the most important markers of adverse outcomes in SARS-CoV-2 infection. As flavonoids can regulate the secretion of pro-inflammatory cytokines and inflammation is the core of the etiopathogenesis of COVID-19 disease, the possibility of these compounds acting as modulators of inflammatory responses and substances alleviating the symptoms of the disease is considered [335,347,348]. For example, a randomized, double-blind, placebo-controlled study where patients who had been diagnosed with COVID-19 received 160 mg of Nano-curcumin in four 40 mg capsules daily for 14 days, was carried out. It confirmed that the administration of Nano-curcumin causes a reduction in the secretion of IL-1β and IL-6. The levels of pro-inflammatory cytokines were significantly higher in the patients with COVID-19 when compared to the healthy control group (Unpaired T-test, *p* = 0.0001 for IL-1β and *p* = 0.0003 for IL-6). Cytokine secretion for Nano-curcumin-treated COVID-19 patients was reduced in comparison to placebo group (Paired T-test, *p* = 0.0082 for IL-1β and *p* = 0.0038 for IL-6) [347]. The anti-inflammatory effect of luteolin (**1**) in IL-1β-stimulated rat OA chondrocytes in vitro and its protective role in a MIA-induced model of OA in vivo were also confirmed [349]. Among other flavonoids modulating the production of inflammatory mediators, apigetrin (a glucoside conjugate of apigenin) reducing IL-1β, TNF-α, IL-6 or catechin (**13**) and glycosylated derivatives of quercetin (**3**), reducing the production of TNF-α, IL-1β, IL-6 and IL-8 have also been described [335,350,351].

The potential of flavonoids as natural substances supporting the prevention of SARS-CoV-2 infection or alleviating the course of COVID-19, due to their extensive biological activity seems to be quite high, although it requires further confirmation.

## 9. Conclusions

In this paper, we have dealt with flavonoids, chromones and coumarins which are well-known plant compounds. These compounds have many similarities related to the main structure and origin. They also have similar biological properties that allow them to be used in the medical and pharmaceutical fields. In this review, we focus on the antioxidant and anti-inflammatory activities of flavonoids, coumarins and chromones, which are characteristic of these compounds and mainly depend on the substitution pattern. In addition, we looked at their metal ion complexes and compared their antioxidant activity with free ligands. The diversity of flavonoid, chromone and coumarin complexes is evident, as well as the involvement of different ligands and coordination modes. Most complexes are in the bidentate *N*,*O* mode, while the tridentate *N*,*S*,*O* mode is characteristic of coumarins. As we all know, antioxidant properties are important for maintaining good health, as many diseases are due to a lack of balance between free radicals and antioxidants. This is especially true of diseases associated with inflammation and cancer. The strength of free radical inhibition depends on the chemical structure and substitution scheme, as well as the functional groups that affect the possibility of binding with metal ions. Therefore, we distinguish different binding motifs that also affect the antioxidant potential of these compounds. Flavonoids, coumarins and chromones are good antioxidants, but the activity depends on the chemical structure. Especially flavonoids with catechol group are good metal chelators and radical scavengers. To study the antioxidant activity of the compounds, the ABTS, DPPH, CRAC, FRAP, FTC and TBA methods were used, and in particular the DPPH method was the most frequently chosen method to determine the antiradical potential of the described compounds. It is also worth mentioning that the scavenging activity of flavonoids was higher after complexation with metal ions. Among the free flavonoids, quercetin has the best antioxidant properties. The other flavonoids, as well as coumarins and chromones, have moderate scavenging activity. Currently, studies are being conducted against COVID-19 using a group of phytochemicals that have been discussed here. Although the group of phytochemicals is diverse, a small number of them have been qualified for clinical trials and are commercially available. To improve the knowledge of natural products and their pharmacological properties, isolation, synthesis, structure-activity relationship, identification of their molecular mechanism and evaluation of biological properties should be carried out.

## Figures and Tables

**Table 1:** TEC_50_ and AE values, scavenging ratio of DPPH and antioxidant activities classification [246].

Compound	TEC_50_ [min]	AE (10^4^)	Classification According to AE	DPPH Radical Scavenging Activities [1 × 10^−4^ M]
Quercetin (**3**)	4.52	7.95	High	61.83
[Cu_2_(L^1^)(H_2_O)_4_]Cl_2_	3.78	11.56	Very high	63.15
[Fe_2_(L^1^)(H_2_O)_8_]Cl_2_	3.24	14.67	Very high	63.94
[Al_2_(L^1^)(H_2_O)_8_]Cl_2_	1.85	32.33	Very high	65.31
[Zn_2_(L^1^)(H_2_O)_4_]Cl_2_	2.40	21.70	Very high	64.24
Rutin (**26**)	62.73	0.09	Low	14.66
[Cu_3_(L^2^)(H_2_O)_6_]Cl_2_	18.04	1.10	Medium	24.70
[Fe_3_(L^2^)(H_2_O)_12_]Cl_2_	13.85	1.54	Medium	25.54
[Al_3_(L^2^)(H_2_O)_12_]Cl_2_	9.15	2.91	Medium	28.19
[Zn_3_(L^2^)(H_2_O)_6_]Cl_2_	11.96	1.96	Medium	26.57
Galangin (**27**)	44.11	0.16	Low	29.17
[Cu(L^3^)(H_2_O)_2_]Cl	10.07	2.6	Medium	30.76
[Fe(L^3^)(H_2_O)_4_]Cl	9.10	3.06	Medium	30.82
[Al(L^3^)(H_2_O)_4_]Cl_2_	5.20	6.48	High	33.49
[Zn(L^3^)(H_2_O)_2_]Cl	6.93	4.48	Medium	32.12
Catechin (**13**)	18.88	1.31	Medium	48.96
[Cu(L^4^)(H_2_O)_6_]Cl	15.59	1.81	Medium	51.01
[Fe(L^2^)(H_2_O)_12_]Cl	13.39	2.16	Medium	51.27
[Al(L^4^)(H_2_O)_12_]Cl_2_	8.65	4.08	Medium	54.34
[Zn (L^4^)(H_2_O)_6_]Cl	10.92	2.93	Medium	52.19

**Table 2:** Substitution scheme of compounds **36**–**41**.

Compound		Substituent	
		R_1_	R_2_
**36**	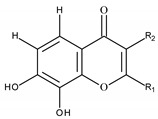	3′-(CF_3_)-Phenyl	3″-(CF_3_)-benzoyl
**37**		3′-(Cl)-Phenyl	3″-(Cl)-benzoyl
**38**		3′-(OCH_3_)-Phenyl	3″-(OCH_3_)-benzoyl
**39**		4′-(F)-Phenyl	4′-(F)-benzoyl
**40**		4′-(NO_2_)-Phenyl	4′-(NO_2_)-benzoyl
**41**		4′-(OCH_3_)-Phenyl	4′-(OCH_3_)-benzoyl

## Supplementary Materials

The following are available, Table S1: Antioxidant activity of chromones **36**–**41**, Table S2: CRAC values for morin (**28**), quercetin (**3**), fisetin (**34**), catechin (**13**), chrysin (**32**) and their complexes with Fe(II) ions, Table S3: IC_50_ values for DPPH free radical savenging, total reductive capability and ferrous ion chelating of compounds. Table S4: Percent of inhibition of 4H_3_NC and its complexes with Fe(II), Ni(II), Zn(II), Cu(II) ions.
