# Supplementary material for: Overview of the Antioxidant and Anti-Inflammatory Activities of Selected Plant Compounds and Their Metal Ions Complexes
Source: Molecules. 2021 Aug 12;26(16):4886. doi: 10.3390/molecules26164886 (PMC8398118; doi:10.3390/molecules26164886)
Supplement: Supplementary file 1 [file molecules-26-04886-s001.zip › molecules-1298406-SI.pdf]

Supporting materials

Overview of the antioxidant and antiinflammatory activities of selected plant compounds and their metal ions complexes

Paulina Mucha*[1], Anna Skoczyńska[2], Magdalena Małecka[3], Paweł Hikisz[4], Elzbieta Budzisz[1*]

[1] Department of the Chemistry of Cosmetic Raw Materials, Faculty of Pfarmacy, Medical University of Łódź, Muszyńskiego 1, 91-419, Poland,
[2] Department of Pharmacology, School of Pharmacy with the Division of Laboratory Medicine in Sosnowiec, Medical University of Silesia, Katowice, Sosnowiec 41-200, Poland anna_sko@onet.pl
[3] Department of Physical Chemistry, Faculty of Chemistry University of Lodz, Pomorska 163/165, 90-236, Łódź, Poland magdalena.malecka@chemia.uni.lodz.pl
[4] Department of Molecular Biophysics, Faculty of Biology and Environmental Protection, University of Lodz, Pomorska 141/143, 90-236 Lodz, Poland, pawel.hikisze@biol.uni.lodz.pl
* Correspondence: elzbieta.budzisz@umed.lodz.pl, paulina.mucha@umed.lodz.pl

Table S1. Antioxidant activity of chromones **36-41**.

Compound	DPPH IC_{50} [μg/ml]	Fe^{2+} ion chelating activity, IC_{50} [μg/ml]	FTIC	TBA
**36**	49.5±0.02	60.05±0.31	94.78±0.03	92.26±0.54
**37**	11.82±0.04	140.46±0.60	88.17±0.30	86.58±0.19
**38**	10.24±0.15	146.69±1.44	93.95±0.42	93.25±1.76
**39**	13.95±0.03	133.86±1.62	94.28±0.23	90.96±1.12
**40**	11.49±0.17	81.67±1.18	93.42±0.19	90.56±0.16
**41**	12.15±0.07	82.35±1.40	88.92±0.47	90.51±1.58

Table S2. CRAC values for morin (**28**), quercetin (**3**), fisetin (**34**), catechin (**13**), chrysin (**32**) and their complexes with Fe(II) ions.

Name of compound	CRAC value x10^6 ([Ce^{3+}]/molxl^{-1}
Morin (**28**)	666.28±9.84
Fe(II)-morin	768.01±1.92
Quercetin (**3**)	476.20±7.59
Fe(II)-quercetin	628.13±11.36
Fisetin (**34**)	341.04±10.02
Fe(II)-fisetin	436.16±6.01
Catechin (**13**)	185.16±3.22

Fe(II)-catechin	188.55±13.32
Chrysin (**32**)	130.44±3.18
Fe(II)-chrysin	140.84±10.94

Table S3. IC_{50} values for DPPH free radical savenging, total reductive capability and ferrous ion chelating of compounds.

Compound	R	R_1	DPPH (IC_{50}) [μg/ml]	Total reductive capability, IC_{50} [μg/ml]	Fe^{2+} ion chelating activity, IC_{50} [μg/ml]
**42**	H	H	64.75 ±0.11	73.09±0.11	65.95±0.11
**43**	Br	H	107.7±0.32	108.17±0.31	105.01±0.31
**44**	H	4-Cl	70.14±0.10	84.21±0.29	93.94±0.29
**45**	Br	4-Cl	185.24±0.15	217.20±0.21	189.29±0.21
**46**	H	4-CH_3	56.19±0.18	62.57±0.15	53.75±0.15
**47**	Br	4-CH_3	59.76±0.21	69.17±0.20	59.45±0.20
**48**	H	4-OCH_3	54.14±0.13	58.01±0.20	56.89±0.20
**49**	Br	4-OCH_3	57.73±0.13	65.52±0.17	62.30±0.17
**50**	H	4-NO_2	92.15±0.17	157.96±0.13	117.36±0.13
**51**	Br	4-NO_2	101.41±0.13	289.02±0.13	153.87±0.13
**52**	H	4-F	88.29±0.21	92.59±0.13	70.76±0.13
**53**	Br	4-F	141.56±0.13	133.90±0.13	131.02±0.13
**Standard a. b, c**			46.95±0.17	50±0.15	44.11±0.15

a Std-BHT is used as standard for DPPH radical scavenging activity.
b Std-BHA is used as a standard for reductive capability
c Std-EDTA is used as a standard for Fe^{2+} ion chelating activity.

Table S4. Percent of inhibition of $4H_3NC$ and its complexes with Fe(II), Ni(II), Zn(II), Cu(II) ions.

Compounds	Inhibitions %
$4H_3NC$	63.7
$4H_3NCFe$	0
$4H_3NCNi$	0
$4H_3NCZn$	Not measured
$4H_3NCCu$	91.2
